# The *nSMase2/Smpd3* gene modulates the severity of muscular dystrophy and the emotional stress response in *mdx* mice

**DOI:** 10.1186/s12916-020-01805-5

**Published:** 2020-11-19

**Authors:** Yasunari Matsuzaka, Jun Tanihata, Yoshiko Ooshima, Daisuke Yamada, Masayuki Sekiguchi, Shouta Miyatake, Yoshitsugu Aoki, Mika Terumitsu, Ryu Yashiro, Hirofumi Komaki, Akihiko Ishiyama, Yasushi Oya, Yukiko U. Inoue, Takayoshi Inoue, Shin’ichi Takeda, Kazuo Hashido

**Affiliations:** 1 Administrative Section of Radiation Protection, National Institute of Neuroscience, National Center of Neurology and Psychiatry, Kodaira, Tokyo Japan; 2 Medical Molecular Informatics, Meiji Pharmaceutical University, Noshio, Kiyose, Tokyo Japan; 3 Department of Molecular Therapy, National Institute of Neuroscience, National Center of Neurology and Psychiatry, Kodaira, Tokyo Japan; 4 Department of Cell Physiology, The Jikei University School of Medicine, Tokyo, Japan; 5 Department of Degenerative Neurological Diseases, National Institute of Neuroscience, National Center of Neurology and Psychiatry, Kodaira, Tokyo Japan; 6 Laboratory of Pharmacology, Department of Pharmacy, Faculty of Pharmaceutical Sciences, Tokyo University of Science, Noda, Chiba Japan; 7 Department of Child Neurology, National Institute of Neuroscience, National Center of Neurology and Psychiatry, Kodaira, Tokyo Japan; 8 Department of Neurology, National Institute of Neuroscience, National Center of Neurology and Psychiatry, Kodaira, Tokyo Japan; 9 Department of Biochemistry and Cellular Biology, National Institute of Neuroscience, National Center of Neurology and Psychiatry, Kodaira, Tokyo Japan

**Keywords:** Duchenne muscular dystrophy, Neutral sphingomyelinase 2/sphingomyelin phosphodiesterase 3, CRISPR-Cas9, Inflammatory cytokine, Monocytes/macrophages, Membrane permeability, Muscle performance, microRNA, Brain-derived neurotrophic factor, Anxiety behavior

## Abstract

**Background:**

Duchenne muscular dystrophy (DMD) is a progressive, degenerative muscular disorder and cognitive dysfunction caused by mutations in the dystrophin gene. It is characterized by excess inflammatory responses in the muscle and repeated degeneration and regeneration cycles. Neutral sphingomyelinase 2/sphingomyelin phosphodiesterase 3 (nSMase2/Smpd3) hydrolyzes sphingomyelin in lipid rafts. This protein thus modulates inflammatory responses, cell survival or apoptosis pathways, and the secretion of extracellular vesicles in a Ca^2+^-dependent manner. However, its roles in dystrophic pathology have not yet been clarified.

**Methods:**

To investigate the effects of the loss of nSMase2/Smpd3 on dystrophic muscles and its role in the abnormal behavior observed in DMD patients, we generated *mdx* mice lacking the *nSMase2/Smpd3* gene (*mdx:Smpd3* double knockout [DKO] mice).

**Results:**

Young *mdx:Smpd3* DKO mice exhibited reduced muscular degeneration and decreased inflammation responses, but later on they showed exacerbated muscular necrosis. In addition, the abnormal stress response displayed by *mdx* mice was improved in the *mdx:Smpd3* DKO mice, with the recovery of brain-derived neurotrophic factor (Bdnf) expression in the hippocampus.

**Conclusions:**

nSMase2/Smpd3-modulated lipid raft integrity is a potential therapeutic target for DMD.

## Background

Duchenne muscular dystrophy (DMD) is an X-linked, recessive, inherited, and debilitating disorder affecting one in 3500 males in Japan. It is caused by loss-of-function mutations in the dystrophin gene on chromosome Xp21 [1]. Disruption of the dystrophin–glycoprotein complex (DGC) on the cell membrane causes cytosolic Ca^2+^ influx, resulting in protease activation, mitochondrial dysfunction, progressive myofiber degeneration, chronic inflammation, muscle wasting, and fragility. The latter phenomena are caused by the replacement of functional myofibers with fibrotic connective tissue and adipose tissue [2–5]. However, the myofiber-specific loss of dystroglycan (DG), a DGC component, in mice does not result in dystrophia-like muscle degeneration, but mice with stem cell-specific deletion of DG do show markedly delayed muscle regeneration [6, 7]. This suggests that myofiber instability is not the only cause of dystrophic degeneration, but rather that the phenotype might be caused by multiple factors, including stem cell and myofiber functions.

In addition to the function of dystrophin in the structural integrity of myofibers described above, a novel function of asymmetric cell division in satellite cells (SC) has been revealed, in which SCs lacking dystrophin show a marked increase in abnormal nonpolarized mitotic divisions and reduced asymmetric cell divisions and myogenic progenitors [7–9]. Thus, the continuing cycles of degeneration and regeneration in the initial stages of the dystrophic pathology exacerbate the phenotype. This exacerbation is thought to be caused by the misregulation of SC fate between differentiation and self-renewing proliferation during the regeneration of degenerated muscles [10, 11]. However, the mechanisms by which SC dysfunction is involved in muscular dystrophy have not yet been elucidated. In addition to muscle degeneration, the loss of dystrophin in the brain has often been associated with nonprogressive cognitive deficits, behavioral disabilities, and enhanced fearfulness [12–14]. However, an effective treatment for these abnormalities has not yet been established.

Neutral sphingomyelinase 2/Sphingomyelin phosphodiesterase 3 (nSMase2/Smpd3) is a membrane-associated enzyme that hydrolyzes sphingomyelin and affects membrane trafficking, receptor clustering, and signal transduction [15, 16]. nSMase2/Smpd3 is activated by inflammatory cytokines such as TNF-alpha, IL-1beta, and interferon-gamma (IFN-gamma), and it in turn activates caspase-12 and calpain in a Ca^2+^-dependent manner [17–19]. In addition, nSMase2/Smpd3 deficiency reduces the inflammatory response through decreased macrophage infiltration and lipid deposition [20]. However, the involvement of nSMase2/Smpd3 in muscular dystrophy and the abnormal behavior observed in DMD patients has not yet been elucidated.

In this study, we test the hypothesis that the pathogenesis in the dystrophic muscles and brains of *mdx* mice is affected by the nSMase2/Smpd3 protein through an inflammation response, as well as regeneration, differentiation, and signaling pathways. Deletion of the *nSMase2/Smpd3* gene from *mdx* mice resulted in decreased inflammation and increased muscle regeneration in the skeletal muscle (SM) in early stages of the dystrophic process, but caused adverse effects in later stages. Furthermore, loss of the *nSMase2/Smpd3* gene in *mdx* mice suppressed abnormal emotional behavior, such as the stress-induced anxiety response, as well as the recovery of hippocampal *Bdnf* expression. Thus, these findings suggest that the nSMase2/Smpd3 protein is a potential therapeutic target for muscular dystrophy and abnormal behavior.

## Methods

### Cell culture

The murine skeletal myoblast C_2_C_12_ cell line were maintained in a proliferation medium that Dulbecco’s modified Eagle’s medium (DMEM; Sigma–Aldrich, St. Louis, MO, USA) supplemented with 10% (v/v) fetal bovine serum (Cell Culture Technologies, Lugano, Switzerland) and 1% (v/v) penicillin/streptomycin (Wako Pure Chemical Industries, Osaka, Japan) at 37 °C in controlled humidified air with 5% CO_2_ in six-well plates (BM Equipment, Tokyo, Japan). Before reaching confluency, the cells were rinsed with PBS, and the proliferation medium was changed to a differentiation medium (DMEM containing 2% heat-inactivated horse serum (HS) and 1% penicillin/streptomycin) until myotube formation was completed.

Dystrophin-deficient H2K myoblasts, which were derived from H-2 kb-tsA58 transgenic mice [21], were seeded at a density of 5 × 10^4^ cells/well in a 75 cm^2^ flask and grown at 33 °C in DMEM with GlutaMAX, IFN-gamma at a concentration of 20 U/mL, and 20% (v/v) fetal bovine serum [22]. After treatment, the cells were differentiated into myotubes by incubating them in DMEM with GlutaMAX containing 5% (v/v) HS at 37 °C. At this plating density, the cells must be passaged once or twice a week, when they reach approximately 10^4^ per cm^2^, and the medium must be changed twice weekly to fresh medium and fresh IFN-gamma. Four days after the initiation of differentiation, which is induced by a high cell density (≥ 10^4^ per cm^2^), the myotubes were used for experiments [23].

### Extraction and quantification of transcripts

As described previously [23], for total RNA extraction, differentiated C_2_C_12_ myotube cells at the indicated times and mouse tissue samples were homogenized. DNA-free RNA was obtained by using the Purelink total RNA extraction kit (Ambion, Austin, TX, USA) according to the manufacturer’s instructions. Specific complementary DNA (cDNA) was synthesized from the purified total RNA with random primers, using the High-Capacity cDNA Reverse Transcription Kit (Applied Biosystems, Foster City, CA, USA). Real-time PCR was performed by the StepOne Real-Time Polymerase Chain Reaction (PCR) System (ABI, Foster City, CA, USA) with gene-specific primers, according to the manufacturer’s instructions (Additional file 1: Table S1). Relative expression of each genes was calculated using SDS 2.1 real-time PCR data analysis software (ABI) and 2^−ΔΔCt^ method. Beta-actin and glyceraldehyde-3-phosphate dehydrogenase (*gapdh*) were used as reference genes for normalization. The gene expression data from triplicate data per one sample are presented as median ± standard error of the mean (SEM).

### Western blotting analysis

The cell lysates were prepared from the differentiated C_2_C_12_ cells and mouse tissues on ice in lysis buffer (50 mM Tris [pH 7.5], 150 mM NaCl, 0.5% Nonident P-40, and protease inhibitor cocktail). Expression of proteins was analyzed by 12% sodium dodecyl sulfate-polyacrylamide gel electrophoresis (SDS-PAGE) using a 12% mini protean precast gel (Bio-Rad Laboratory, Hercules, CA, USA). The protein band was transferred to a polyvinylidene difluoride membrane (Merck Millipore, Billerica, MA, USA). Then, the membrane was incubated in gene-specific antibodies (1:1000 in a mixture of tris-buffered saline and polysorbate 20 [TBST]) (1:1000 in TBST) for 1 h at RT with gentle shaking. Then, the membrane was washed for 3 times with TBST having 0.5% milk. An anti-rat immunoglobulin G (IgG)-conjugated to horseradish peroxidase (HRP) antibody (1:1000 in TBST) was used for visualization as secondary antibody (1:1000 in TBST). The membrane was incubated for 1 h at RT with gentle shaking. Then, the membrane was washed for 3 times with TBST having 0.5% milk. For signal detection of specific band, the membrane was analyzed on the LAS-3000 Imager (Fujifilm Corporation, Tokyo, Japan) after reaction using antibodies coupled to HRP and ECL Prime Detection Reagent (GE Healthcare, Chicago, IL, USA). The primary antibodies used in this study were as follows: anti-Smpd3 (Eurofins Genomics, Tokyo, Japan); anti-BDNF [EPR1292] (ab182199; Abcam, Cambridge, UK) which is a synthetic peptide immunogen within human BDNF, covering aa 150 to the C-terminus; anti-caveolin-3 (Eurofins Genomics); anti-p65 [E379]; anti-phospho-p65 [EP2294Y] (Abcam); anti-Gapdh (BioVision, Milpitas, CA, USA); and anti-dystrophin [NCL-DYS2] (Novocastra Laboratories, Newcastle upon Tyne, UK).

### Measurement of SMase enzymatic activity

SMase enzymatic activity was measured using the Sphingomyelinase Activity Colorimetric Assay Kit according to the manufacturer’s protocol (BioVision). Briefly, GAS muscles were removed from wt, *mdx*, and *mdx:Smpd3*^*−/−*^ (#238 and #11) mice, and then SMase Assay Buffer and SMase Extraction Detergent with Protease Inhibitor Cocktail (Promega, Madison, WI, USA) were added. To extract the tissue lysate, GAS muscles were homogenized on ice, and supernatants were collected after centrifugation at 10,000×*g* for 5 min. Next, 5 μL of supernatant was added to a 96-well plate with 45 μL of SMase Assay Buffer. The reaction mix, containing 32 μL of SMase Assay Buffer, 2 μL of SMase Enzyme Mix I, 10 μL of SMase Enzyme Mix II, 4 μL of SMase Substrate, and 2 μL of SMase Probe, was added to each well containing the sample solution. After mixing well, the reaction solution was incubated for 30 min at 37 °C and the absorbance at 570 nm was measured.

### Generation of *nSMase2/Smpd3* knockdown mice

Under specific pathogen-free (SPF) condition, all mice lines used in this study were maintained at 21 °C on a 12:12 h light to dark cycle at the National Institute of Neuroscience (NCNP), Japan, and treated in accordance with the guidelines provided by the Ethics Committee for the Treatment of Laboratory Animals of the National Center of Neurology and Psychiatry (approval ID: 2015006), which has adopted the three fundamental principles of replacement, reduction, and refinement. Cages were always enriched with crinkle paper and animals were allowed to drink water and eat food ad libitum. Consistent with the protocol stipulated by the research permit, all efforts were made to minimize the suffering and discomfort experienced by the animals. To design sgRNAs against the *nSMase2/Smpd3* gene, the Clustered Regularly Interspaced Short Palindromic Repeats (CRISPR) Design Tool (http://crispr.mit.edu) was used to search for candidate nucleotide sequences, and we selected three candidate target sequences based on the prediction scores of the CRISPR Design Tool (Additional file 2: Table S2). The plasmids expressing CRISPR-associated protein 9 (Cas9) and the sgRNAs were constructed by ligating three oligonucleotide pairs against each sgRNA with the BbsI-digested pX330 plasmid (Addgene, Watertown, MA, USA) according to the manufacturer’s instructions [24]. In brief, to prepare for the ligation of the sgRNAs into the BbsI site of the pX330 plasmid, three oligonucleotide pairs were added using four tagged nucleotide sequences, i.e., 5′-cacc-3′ at the 5′-end of the forward oligonucleotide and 5′-caaa-3′ at the 3′-end of the reverse oligonucleotide, together with 0.1 μM of forward and reverse oligonucleotides in tris-ethylenediaminetetraacetic acid (TE) buffer, and the oligonucleotides were annealed using a thermal cycler, as follows: one cycle of 95 °C for 5 min, 60 °C for 5 min, and 25 °C for 60 min. The ligation solution was prepared as follows: annealed oligonucleotides, BbsI-digested pX330, and Ligation high buffer, and this was incubated for 1 h at 16 °C. The plasmid DNA-ligated sgRNA was transformed into a competent *Escherichia coli* strain (Competent Quick DH5alpha, TOYOBO, Tokyo, Japan), and incubated on Luria-Bertani (LB) containing 100 μg/mL ampicillin at 37 °C for 14 h.

Individual colonies on the plate were picked up and incubated in LB liquid culture containing 100 μg/mL ampicillin with shaking overnight at 37 °C for 16 h. The plasmid DNA was purified from the liquid cultures using a spin column from a commercially available kit (PureLinkR HiPure Plasmid Maxiprep Kit, Invitrogen, Carlsbad, CA, USA) according to the manufacturer’s instructions, and integration of the sgRNA into the BbsI site of the pX330 vector was confirmed by direct sequencing. SgRNA-pX330 plasmid DNA in their circular form were injected directly into the pronuclei of zygotes collected from the oviducts of wt female B6C3F1 mice mated to wt male B6C3F1 mice, to reduce their integration into the host mouse genome, although the transgenic efficiency with the circular form of the plasmid DNA is approximately 10 times lower than that of the linear form [24, 25].

To screen for target mutant mice, genomic DNA was extracted from tissue samples taken from the tails of pups that developed from the microinjected eggs, by incubating them in lysis buffer at 50 °C for 17 h followed by phenol/chloroform purification. The extracted DNA was then genotyped by PCR amplification, the amplified PCR fragments were purified using DNA purification spin columns, and nucleotides were determined by sequencing with the indicated primer set (Additional file 3: Table S3). To determine the integration of Cas9 nucleotides into the genome DNA of the mice targeted by the sgRNAs, PCR amplification was performed using a pair of primers for the Cas9 nucleotide sequence. To check for off-target effects, a homology search for the three sgRNAs with a mouse genome sequence was performed using BLAST (https://blast.ncbi.nlm.nih.gov/Blast.cgi). The resulting matching genomic sequences were amplified and their nucleotide sequences were determined via PCR amplification and sequenced using region-specific primer sets. To generate *nSMase2/Smpd3*-null, dystrophin-deficient DKO mice, we crossed *nSMase2/Smpd3* KO mice with *mdx* mice. The genetic background of the *mdx:nSMase/Smpd3* DKO mice used in this study is a mix that was created by mating mice from the B6C3F1 and B6j backgrounds and used after 4–5 generations of inbreeding.

### Tissue preparation

Mice were sacrificed by cervical dislocation. Body and wet muscle weight were measured. The TA, GAS, and diaphragm muscles were collected using standard dissection methods, as described previously [26]. Some muscle samples were frozen in isopentane cooled by liquid nitrogen for histological analysis, and the remaining muscle samples were frozen in liquid nitrogen for RNA or protein isolation, and stored at 80 °C as described previously [26, 27]. Transverse cryosections (20 μm thick) of each muscle were stained with H&E, as described previously [28].

### Patients

A total of 119 unrelated Japanese patients with DMD, BMD, myotonic dystrophy 1, distal myopathy with rimmed vacuoles (DMRV), facioscapulohumeral muscular dystrophy (FSHD), limb-girdle muscular dystrophy (LGMD), or limb-girdle muscular dystrophy 2B (LGMD2B) were participated in this study (Additional file 4: Table S4). Informed consent form with the patient’s signature was obtained from all participants after the details of the study had been explained to them and prior to the collection of peripheral blood. The protocol was approved by Research Ethics Committee of the NCNP (approval ID: A2011-113), in accordance with the regulations of the Declaration of Helsinki. All results were treated with standard medical confidentiality, and confidentially was maintained to the extent stipulated by the law.

### Measurement of CK activity

From the blood samples, the supernatant was removed by microcentrifugation after incubation at room temperature. Serum CK assays were carried out using a commercially available Fuji Dri-Chem system (Fujifilm Medical, Tokyo, Japan) according to the manufacturer’s protocol, as described previously [23]. Serum (10 μL) was incubated at 37 °C on a Fuji Dri-Chem slide, and the dye absorbance was measured spectrophotometrically for 5 min at 540 nm. The values were calculated according to the installed formula, and data are expressed as units per liter (U/L).

### Muscle Evans blue dye uptake experiments

To assess muscle damage, EBD (Nacalai Tesque, Tokyo, Japan) was dissolved in phosphate buffered salts (PBS) and sterilized by filtration. Twenty-four hours before sacrifice, mice were injected intraperitoneally with 1% EBD as described previously [29]. The muscle tissues were collected, and then frozen in melting isopentane. The sectioned muscle was created, and incubated in acetone (ice-cold) for 10 min. After wash the sections three times for 10 min with PBS, the flat-mount muscle was created by Vectashield mounting medium. Fluorescence microscopy was used for evaluation of the presence of EBD in myofibers.

### Treadmill and grip strength tests

The treadmill muscle performance test was performed as described previously [30].

Briefly, mice were placed on a motor-driven flat MK-680S treadmill system (Muromachi Kikai, Tokyo, Japan) and forced to run for 5 min at a speed of 5 m/min. After 5 min, the speed was accelerated by 1 m/min every min. The test was stopped when the mouse was exhausted and did not attempt to remount the treadmill, and the time to exhaustion was recorded.

The forelimb grip strength of the mice was monitored using a grip strength meter (MK-380 M, Muromachi Kikai) with the investigator blinded to genotype. The mice were held 2 cm from the base of the tail, allowed to grab a woven metal wire with their forelimbs, and were pulled gently in the horizontal plane until they released their grip. The force at the time of release was recorded as peak tension. Five sequential tests were carried out for each mouse, at 5 s intervals. The average peak tension in these attempts was defined as forelimb grip strength.

### Extraction and quantification of miRNA

Total RNA isolation from serum or tissues was performed according to the manufacturer instructions for the PureLinkR RNA Mini Kit (Ambion, Austin, TX, USA) as previously described [31, 32]. The cDNA derived from the total RNA was prepared using a TaqMan miRNA Reverse Transcription (RT) kit (ABI, Foster City, CA, USA) and miRNA-specific stem-loop primers (part of the TaqMan miRNA assay kit; ABI) as previously described [23, 33]. For the real-time PCR of the miRNA, we used individual miRNA-specific primers (part of the TaqMan miRNA assay kit; ABI) with the StepOne Real-Time PCR System (ABI) according to the manufacturer’s protocol. Each miRNA was assayed in triplicate and data are presented as median values with the standard deviation. The relative expression levels for each miRNA were normalized using by endogenous and exogenous controls that are miR-16 and cel-miR-39. SDS 2.1 real-time PCR data analysis software (ABI) was used for the data analysis.

### Cell viability analysis

The C_2_C_12_ or H2K cell line was seeded in 96-well plates (BM Equipment, Tokyo, Japan). Ten microliters of 2-(2-methoxy-4-nitrophenyl)-3-(4-nitrophenyl)-5-(2,4-disulfophenyl)-2H-tetrazolium salt from the Cell Counting Kit-8 (CCK-8) (DOJINDO Laboratories, Kumamoto, Japan) was added to each well to detect cell proliferation and cell toxicity based on the quantity of formazan dye generated by the dehydrogenases in the cells, as described previously [23]. The cells continued to be cultured for 2 h, and then the absorbance at 450 nm of each well was measured using a Model 680-well microplate reader (Bio-Rad Laboratory).

### Proteome profiler cytokine array

The skeletal and diaphragm muscles of *mdx:Smpd3*^*−/−*^ (#238) and *mdx* mice were excised and homogenized in PBS with protease. After homogenization, Triton X-100 was added to a final concentration of 1%. To remove cellular debris, the tissue lysates were centrifuged at 10,000×*g* for 5 min. The protein concentration of the lysates was determined, and the relative expression of the cytokines and chemokines in the lysates was quantified using the Proteosome Profiler Array (Mouse Cytokine Array, Panel A; R&D Systems, Minneapolis, MN, USA) according to the manufacturer’s protocol. Briefly, 2.0 mL of Array Buffer was added into each well of the 4-well multi-dish as a blocking buffer. Each membrane was incubated for 1 h in a well of a 4-well multi-dish plate on a platform shaker. 1.5 μL of reconstituted Mouse Cytokine Array Panel A Detection Antibody Cocktail was added to each prepared sample, and the samples were then incubated at room temperature for 1 h. After blocking, the Array Buffer was aspirated from the wells. The sample/antibody mixtures were placed on the 4-well multi-dish and incubated overnight at 4 °C on a rocking platform shaker. Each membrane was washed thrice with 1× Wash Buffer for 10 min on a rocking platform shaker. The membrane was incubated with diluted Streptavidin-HPR for 30 min at room temperature on a rocking platform shaker. Then, after the membrane had been washed, signal detection was performed using the Chemi Reagent Mix and analyzed on the LAS-3000 Imager (Fujifilm Corporation).

### Measurement of cathepsin B activity using an in vivo imaging system

ProSense 680 is a small peptide substrate for activated cathepsin B (CTSB). When cleaved by the CTSB enzyme, two caged fluorophores are released, with peak excitation at 680 nm and emission in the near-infrared range (700 nm). ProSense 680 (0.75 or 1.5 nmol) was intraperitoneally injected into 5-week-old wt, *mdx*, *mdx:Smpd3*^*−/−*^, and *Smpd3*^*−/−*^ mice, which were then placed on a heating pad under anesthesia to keep their body temperature constant. Imaging was performed using a single excitation/emission filter pair optimal for the wavelength of the probe.

### Apoptosis assay

Caspase-3 and caspase-9 activities were determined using Caspase-3/CPP32 or Caspase-9 colorimetric Assay Kit (BioVision), respectively, according to the manufacturer’s protocol. Briefly, GAS and TA muscles and cerebellum of *mdx:Smpd3*^*−/−*^, *mdx*, and wt mice were homogenized, and then lysis buffer (50 mM Tris [pH 7.5], 150 mM NaCl, 0.5% Nonident P-40, and protease inhibitor cocktail) was added. After incubation on ice for 10 min, the tissue lysates were centrifuged at 10,000×*g* for 5 min for removal of cellular debris. The DEVD-pNA substrate was added into the sample solution adjusted the protein concentration and incubated at 37 °C for 2 h. The absorbance of each well at 405 nm was determined using a Model 680-well microplate reader (Bio-Rad Laboratory).

### Restraint test in mice

Mice were restrained by the experimenter by placing the neck between the thumb and index finger and putting the tail between the third and little fingers [12]. After 10 s, the mouse was released into a cage (24 cm × 17 cm, surrounded by a 12 cm-high wall) containing wood chips (illuminated at 80 lx). A camera on the ceiling of the cage recorded a video and saved it to a personal computer. Locomotion and freezing were calculated from the image files obtained during the 5 min after the restraint using Image OF (O’Hara & Co., Tokyo, Japan), which is a modified version of the public-domain NIH Image program (developed at the US National Institutes of Health and available from http://rsb.info.nih.gov/nih-image/). Complete immobilization of the mouse, except for respiration, was regarded as a freezing response.

### Sample size and statistical power

To determine the effective sample size needed to ensure the required statistical power in tests involving *mdx* and *mdx:Smpd3*^*+/−*^ or *mdx:Smpd3*^*−/−*^ mice and DMD and BMD patients, we calculated sample sizes based on the means of populations 1 and 2 and the common standard deviation, using the Power/Sample Size Calculator (available from https://www.stat.ubc.ca/~rollin/stats/ssize/n2.html) (Additional files 5, 6: Table S5, S6).

## Results

### Expression of *nSMase/Smpd*-family genes during C_2_C_12_ differentiation and generation of *nSMase2/Smpd3* knockdown mice in the *mdx* genetic background

To investigate the expression patterns of *nSMase2/Smpd3* genes in response to the induction of myogenic differentiation in C_2_C_12_ mouse myoblast cells, we quantified the mRNA expression levels of acid sphingomyelinase (*aSMase*)/*Smpd1*, *nSMase*/*Smpd2*, and *nSMase2*/*Smpd3* during the course of C_2_C_12_ differentiation. All three genes were upregulated as early as 4 days after induction with differentiation medium, when the cells became phase-bright fused myotubes, and the upregulated levels were sustained for 6 days (Additional file 7: Fig. S1A). The expression level of the adult myosin heavy chain (*Myh1*) gene was used as a differentiation marker for C_2_C_12_ myoblasts, similar to the expression pattern of *nSMase2/Smpd3* (Additional file 7: Fig. S1A). Western blotting showed that the nSMase2/Smpd3 protein was upregulated until day six (Additional file 7: Fig. S1B). On the other hand, the Bdnf protein was upregulated at day one, peaked on days two and three, then decreased until day six (Additional file 7: Fig. S1B). These findings indicate that nSMase2/Smpd3 is essential for myoblast differentiation.

To define the role of *nSMase2/Smpd3* in the dystrophic process, we generated *nSMase2/Smpd3* DKO mice using the CRISPR-Cas9 system with three single guide RNAs (sgRNAs) located in a large insertion region (LIR) between catalytic domain 1 (CD1) and CD2 of the nSMase2/Smpd3 protein (Additional file 7: Fig. S1C: lower panel). To reduce the possibility of off-target effects, we searched the mouse genome for regions homologous to the three sgRNA target nucleotide sequences using a BLAST search. We examined a total of 82 nucleotide sequences from homologous genomic regions for off-target effects using PCR-amplification and direct sequencing of the genomic DNA of each *nSMase2/Smpd3* DKO mouse line generated (data not shown). In addition, the genomic DNA was analyzed to ensure that the Cas9 transgene (Tg). After the mouse lines with off-target effects or the Cas9 Tg had been eliminated, six independent *nSMase2/Smpd3* DKO mouse lines were established, of which one (#20) had a premature stop codon at the amino acid (AA) at position 224 of the nSMase2/Smpd3 protein, with the last seven AAs included as substitutions (Additional file 7: Fig. S1C and D). The other five lines showed AA deletions within the nSMase2/Smpd3 protein at AA positions 219–220 (#11) or 243–245 (#26), a deletion of AA 246 with a substitution at position 245 from Arg to His (#28), a deletion of AAs 245–246 with a substitution at AA position 244 from Ile to Arg (#30), or a deletion from AA 191–273 with 30 AA insertions (#238) (Additional file 7: Fig. S1C and D).

To generate *nSMase2/Smpd3*-null, dystrophin-deficient double-mutant (DM) mice, we crossed *nSMase2/Smpd3* DKO mice with *mdx* mice (Additional file 8: Fig. S2A and B). These founders were not born in a Mendelian ratio (Additional file 9: Table S7A and B), but there were no differences in organ weights between them and *mdx* mice (data not shown). Immunoblot analysis indicated that the nSMase2/Smpd3 protein was expressed in the gastrocnemius (GAS) muscle of both *mdx* and *mdx:Smpd3*^*−/−*^ DKO (#30) mice at 18 weeks of age (Additional file 10: Fig. S3A), in the cerebellum of wild-type (wt), *mdx*, and *mdx:Smpd3*^*−/−*^ DKO (#238) mice at 14 weeks of age (Additional file 10: Fig. S3B), and in the hippocampus of both *mdx* and *mdx:Smpd3*^*−/−*^ DKO (#238) mice at 5 and 9 weeks of age (Additional file 10: Fig. S3C). In addition, the hippocampi of two of the *mdx:Smpd3*^*−/−*^ DKO mouse lines, namely #11 (at 12 weeks) and #30 (at 18 weeks), expressed nSMase2/Smpd3 proteins, but in mouse line #28 at both 6 and 13 months, and in line #20 at 19 weeks, the specific band for nSMase2/Smpd3 could not be detected (Additional file 10: Fig. S3C). In addition, analysis of lysates of the SM and hippocampi of wt, *mdx*, and *mdx:Smpd3*^*−/−*^ DKO (#238 and #11) mice showed that there was no difference in nSMase enzymatic activity between the #238 line and *mdx* mice in either the SM or the hippocampus (Additional file 10: Fig. S3C). However, the nSMase activity in the #11 line was significantly lower in the SM (Additional file 10: Fig. S3D) and somewhat lower in the hippocampus (Additional file 10: Fig. S3E) than in the #238 line and *mdx* mice. These results indicated that the LIR, in part, regulates nSMase/Smpd3 activity in vivo.

### Deletion of the *nSMase2/Smpd3* gene in *mdx* mice reduces inflammation in dystrophic muscles

**Fig. 1:**
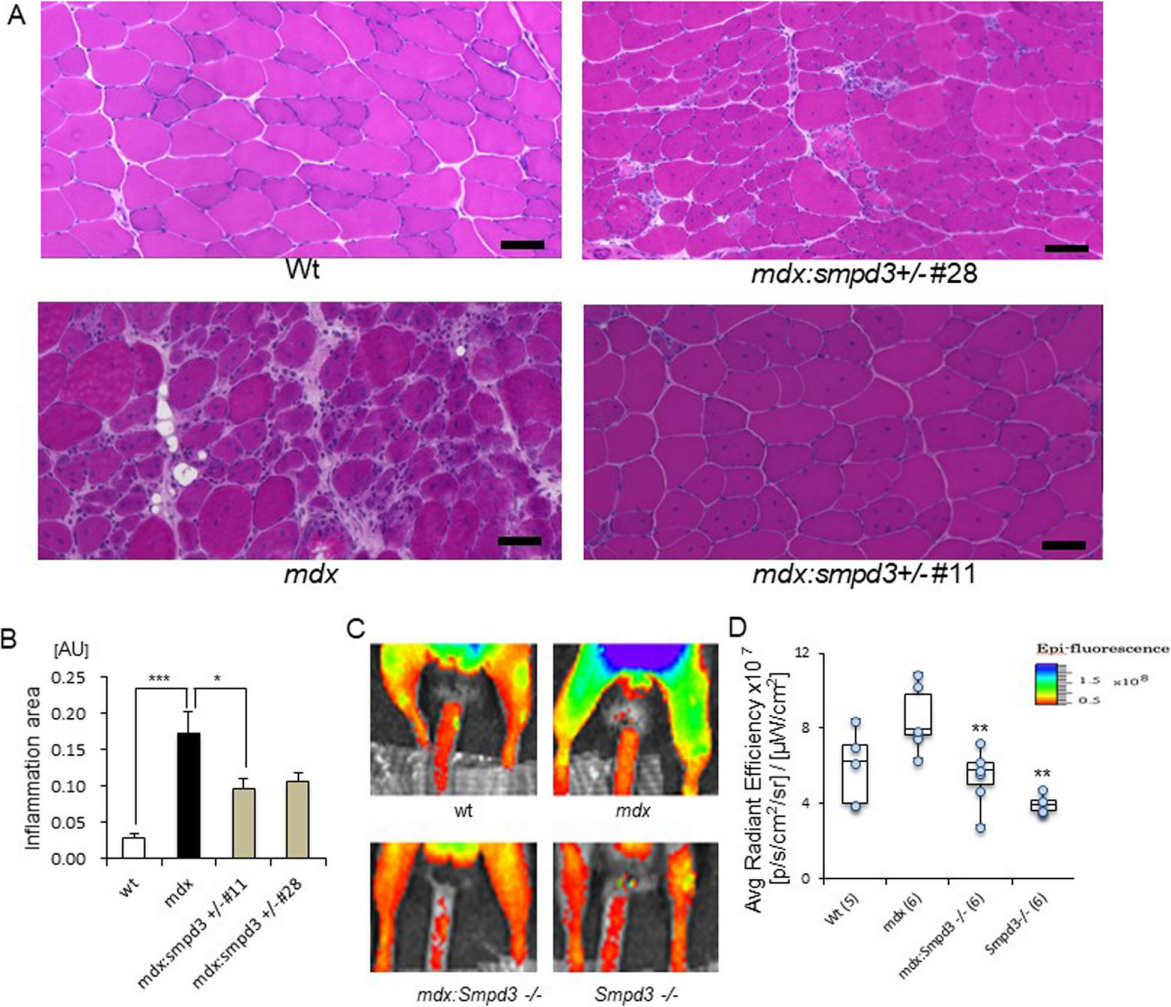
Deletion of the *nSMase2*/*Smpd3* gene attenuates the inflammatory response in *mdx* mice. **a** Representative images of H&E-stained tibialis anterior (TA) muscle sections from wt, *mdx*, and *mdx:Smpd3*^*+/−*^ mice (lines #11 and #28) at 6 weeks of age. Scale bars = 50 μm. **b** The area of the inflammation from the TA sections of wt, *mdx*, and *mdx:Smpd3*^*+/−*^ mice (#11 and #28) were quantitated (*n* = 3 per genotype) (**c**, **d**). Representative images for quantification of inflammation in the hindlimb muscles, measured by cathepsin-B activity via live-animal optical imaging of wt, *mdx*, *mdx:Smpd3*^*−/−*^, and *Smpd3*^*−/−*^ mice (**c**) and quantification of the inflammation intensities (**d**). The numbers of animals used are indicated in parentheses

To characterize the contribution of the *nSMase2/Smpd3* gene to muscular inflammation in dystrophic muscles, cross sections of tibialis anterior (TA) muscles from 6-week-old mice were stained with hematoxylin and eosin (H&E). The area of inflammatory infiltration was significantly smaller in 6-week-old *mdx:Smpd3*^*+/−*^ (#11) mice than in *mdx* mice (Fig. 1a, b, *p* < 0.05). To quantify the inflammation in the limb muscles of live animals, cathepsin-B enzyme activity was measured as an indicator of inflammation. The hindlimb muscles of *mdx* mice exhibited higher levels of cathepsin-B activity than those of wt mice and *mdx:Smpd3*^*−/−*^ mice, and activity levels in the muscles of *Smpd3*^*−/−*^ mice were significantly lower than in wt mice (Fig. 1c, d, *p* < 0.01).

**Fig. 2:**
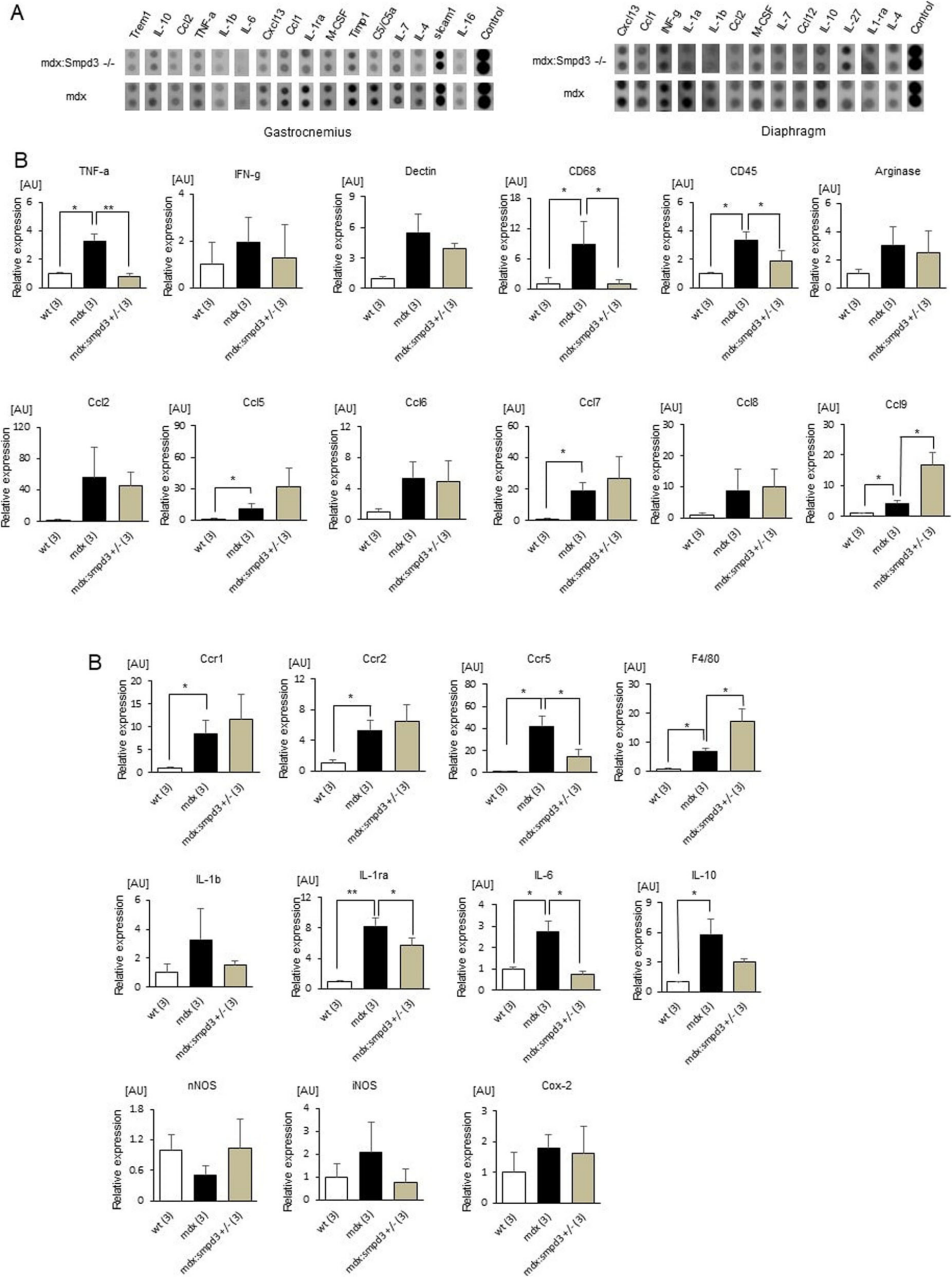
Analysis of inflammation-related gene expression. **a** Expression levels of the cytokines and chemokines in the gastrocnemius (GAS) muscle (left) and diaphragm (right) of 12-week-old *mdx* and *mdx:Smpd3*^*−/−*^ mice. **b** Expression of inflammation-related genes in the GAS of wt, *mdx*, and *mdx:Smpd3*^*+/−*^ mice at 12 weeks of age was measured by real-time RT-PCR (*n* = 3 per genotype). *p < 0.05, ** *p* < 0.01

Next, to analyze the protein expression levels of inflammatory cytokines that can be used as cellular markers, we performed multiple cytokine/chemokine protein array analyses on GAS and diaphragm muscles extracted from *mdx:Smpd3*^*−/−*^ and *mdx* mice (Additional file 11: Fig. S4). The expression of *Trem1* was lower in *mdx:Smpd3*^*−/−*^ mice than in *mdx* mice (Fig. 2a: left). The levels of markers of activated macrophages, such as Ccl2 (M1 and M2a macrophages), Cxcl13 (M1 macrophages), IL-1beta (M1 and M2b macrophages), IL-6 (M1 and M2b macrophages), Ccl1 (M2a and M2b macrophages), IL-1ra (M2a macrophages) and IL-10 (M2a, M2b, M2c, and M2d macrophages), and TNF-alpha (M1, M2b, and M2d macrophages) were lower in *mdx:Smpd3*^*−/−*^ mice than in *mdx* mice (Fig. 2a: left). In the diaphragm muscle, activated macrophage markers such as Cxcl13, Ccl1, IL-1beta, Ccl2, and IL-10 were also lower in *mdx:Smpd3*^*−/−*^ mice than in *mdx* mice (Fig. 2a: right).

**Fig. 3:**
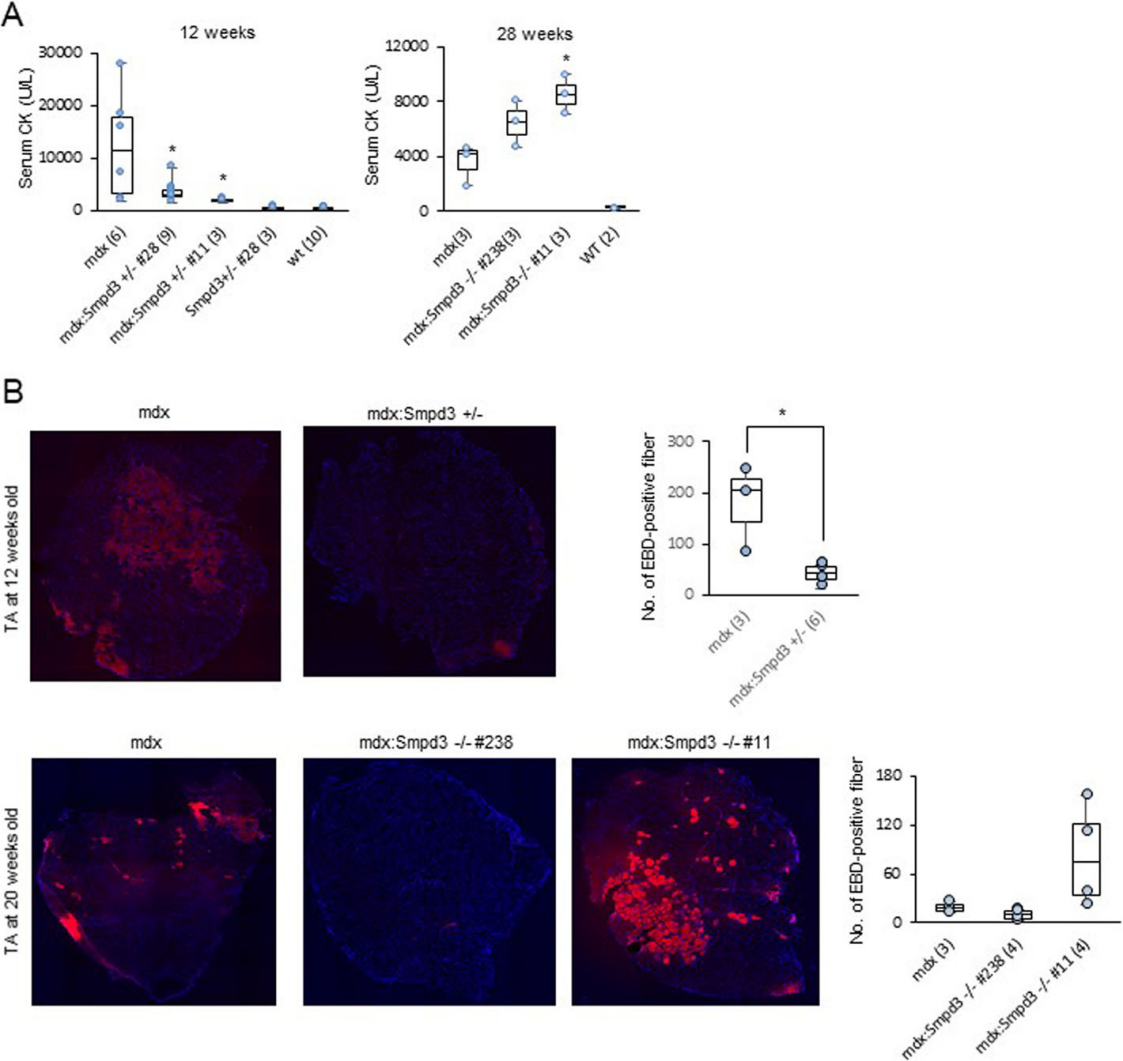
*Smpd3* ablation reduces sarcolemmal instability in the muscles of young *mdx* mice but exacerbates instability in older mice. **a** Serum creatine kinase (CK) levels of *mdx*, *mdx:Smpd3*^*+/−*^, *mdx:Smpd3*^*−/−*^, *Smpd3*^*+/−*^, *Smpd3*^*−/−*^, and wt mice at 12 and 28 weeks of age. **b** Representative Evans blue dye (EBD) uptake in the tibialis anterior (TA) muscle of *mdx* and *mdx:Smpd3*^*+/−*^ mice at 12 weeks of age and *mdx* and *mdx:Smpd3*^*−/−*^ (#11 and #238) mice at 20 weeks of age. The number of EBD-positive fibers per arbitrary unit area was counted (right). The number of animals used is indicated in parentheses. Numbers preceded by # represent mouse lines. *p < 0.05

Next, transcription levels of inflammatory cytokines in *mdx:Smpd3 DKO* and *mdx* mice were analyzed using real-time quantitative polymerase chain reaction (qPCR) analysis. In the GAS of 12-week-old *mdx:Smpd3*^*+/−*^ mice, levels of *TNF-alpha*, *CD68*, *CD45*, *Ccr5*, *IL-1ra*, and *IL-6* were significantly lower than in *mdx* mice (Fig. 3b). However, *Ccl9* and *F4/80* expression levels in the GAS of *mdx:Smpd3*^*+/−*^ mice were significantly higher than in *mdx* mice (Fig. 2b, *p* < 0.05). In addition, the diaphragm muscle of 12-week-old *mdx:Smpd3*^*+/−*^ mice had significantly lower levels of MyoD than that of *mdx* mice (Additional file 12: Fig. Fig. S5 *p* < 0.05). TNF-alpha levels in the GAS of 12-week-old *mdx:Smpd3*^*−/−*^ mice were significantly lower than in *mdx* mice (#238, *p* < 0.01; #11, *p* < 0.001), whereas at 18 weeks old, levels in the #238 line were similar to those in *mdx* mice (Additional file 13: Fig. S6). In addition, IL-6 levels in *mdx:Smpd3*^*−/−*^ (#238 and #11) mice at 12 weeks and the #238 line at 18 weeks were significantly lower than in *mdx* mice (Additional file 13: Fig. S6, *p* < 0.05).

To characterize the inflammatory cell populations within the SM of *mdx* and *mdx:Smpd3*^*−/−*^ mice, we used real-time qPCR to compare the mRNA expression levels of molecular markers of inflammation in the GAS muscles of 12-week-old *mdx* and *mdx:Smpd3*^*−/−*^ (#238 and #11) mice. The expression levels of *CD45* (monocytes) and *Dectin-1* (M2a macrophages) in *mdx:Smpd3*^*−/−*^ (#238) mice were significantly lower than in *mdx* mice (Additional file 13: Fig. S6, *p* < 0.01 and *p* < 0.05, respectively). In addition, the levels of *Fizz/Retnla* (M2a macrophages), *IL-10* (M2a macrophages), and *Ccr5* (monocytes) in *mdx:Smpd3*^*−/−*^ (#238) mice were somewhat lower than in *mdx* mice (Additional file 13: Fig. S6). We also analyzed molecular markers for monocytes/macrophages in the GAS of *mdx:Smpd3*^*−/−*^ mouse lines #238 and #11. The level of *CD11b* (monocytes/macrophages) was significantly lower in the #238 line, but not in the #11 line, than in *mdx* mice (Additional file 13: Fig. S6, *p* < 0.05). In contrast, the expression level of *F4/80* (a mature macrophage marker) in *mdx:Smpd3*^*−/−*^ (#11) mice was approximately 10-fold that in *mdx* mice (Additional file 13: Fig. S6). In addition, expression levels of the *nNOS* gene in the GAS of *mdx:Smpd3*^*−/−*^ (#238) mice at 9 and 12 weeks of age were higher than in *mdx* mice (Additional file 13: Fig. S6, ns and *p* < 0.05, respectively). In addition, expression of the *Cox-2* gene at 12 weeks of age in the GAS of *mdx:Smpd3*^*−/−*^ mice was significantly lower than in *mdx* mice (Additional file 13: Fig. S6, *p* < 0.05).

To assess the effects of nSMase2/Smpd3 on the phosphorylation of Ser536 of p65, we performed a western blot analysis on the GAS of *mdx:Smpd3*^*−/−*^ and *mdx* mice. However, we found no obvious differences in the expression of p65 or phosho-p65 between *mdx* and *mdx:Smpd3*^*−/−*^ mice (data not shown). At 17 weeks, expression of *Foxp3* in the GAS of *mdx:Smpd3*^*−/−*^ mice was significantly lower than in *mdx* mice (Additional file 13: Fig. S6, *p* < 0.01), whereas at 19 weeks it was significantly higher (Additional file 13: Fig. S6, *p* < 0.05). Combined, these data suggest that depletion of nSMase2/Smpd3 may reduce inflammatory cytokine expression in monocytes/macrophages in the dystrophic process.

### Disruption of the *nSMase2/Smpd3* gene attenuates muscle membrane permeability in dystrophic *mdx* mice early on, but exacerbates it later

To assess the potential contribution of *nSMase2/Smpd3* ablation on myofiber membrane permeability in the dystrophic SM of *mdx* mice, we measured the levels of serum creatine kinase (CK) as a serum biomarker for dystrophic muscle in wt, *md*x, and *mdx:Smpd3* DKO mice. In 6-week-old *mdx:Smpd3*^*+/−*^ mice, serum CK levels were approximately 67% lower than those in *mdx* mice (Additional file 14: Fig. S7A). Similarly, at 8–10 weeks of age, serum CK levels in two *mdx:Smpd3*^*+/−*^ mice groups (#20, #28, and #35) and (#11), and *mdx:Smpd3*^*−/−*^ (#11) mice were significantly lower than in *mdx* mice (Additional file 14: Fig. S7B, *p* < 0.05 for *mdx:Smpd3*^*+/−*^ mice #20, #28, and #35, *p* < 0.01 for *mdx:Smpd3*^*+/−*^ mice # 11, and *p* < 0.01 for *mdx:Smpd3*^*−/−*^ mice # 11). We also observed significantly lower levels of serum CK in *mdx:Smpd3*^*+/−*^ (#28 and #11) mice at 12 weeks old (Fig. 3a: left, *p* < 0.01) and *mdx:Smpd3*^*−/−*^ (#30) mice at 18 weeks old (Additional file 14: Fig. S7C, *p* < 0.001) than in *mdx* mice. However, at 28 weeks, we observed somewhat (#238) or significantly (#11; *p* < 0.05) higher serum CK levels in *mdx:Smpd3*^*−/−*^ mice than in *mdx* mice (Fig. 3a: right).

Next, to visualize and quantify the degree of myofiber damage, we analyzed uptake of Evans blue dye (EBD) in the TA hind limb and diaphragm muscles. At 12 weeks of age, there were significantly fewer EBD-positive muscle fibers in the TA of *mdx:Smpd3*^*+/−*^ mice than in *mdx* mice (Fig. 3b: upper, *p* < 0.05), but there was no difference between the diaphragm muscles of *mdx:Smpd3*^*+/−*^ and *mdx* mice (data not shown). The number of EBD-positive muscle fibers in the TA of line #238 *mdx:Smpd3*^*−/−*^ mice at 20 weeks of age was also substantially lower than in *mdx* mice, but it was higher in line #11 *mdx:Smpd3*^*−/−*^ mice (Fig. 3b: bottom). These results suggest that early in life, *nSMase2/Smpd3* ablation may have beneficial effects with respect to myofiber membrane degeneration in *mdx* mice, but that later on it may have adverse effects.

### Genetic ablation of *nSMase2/Smpd3* improves muscle performance in mice with dystrophic phenotypes

**Fig. 4:**
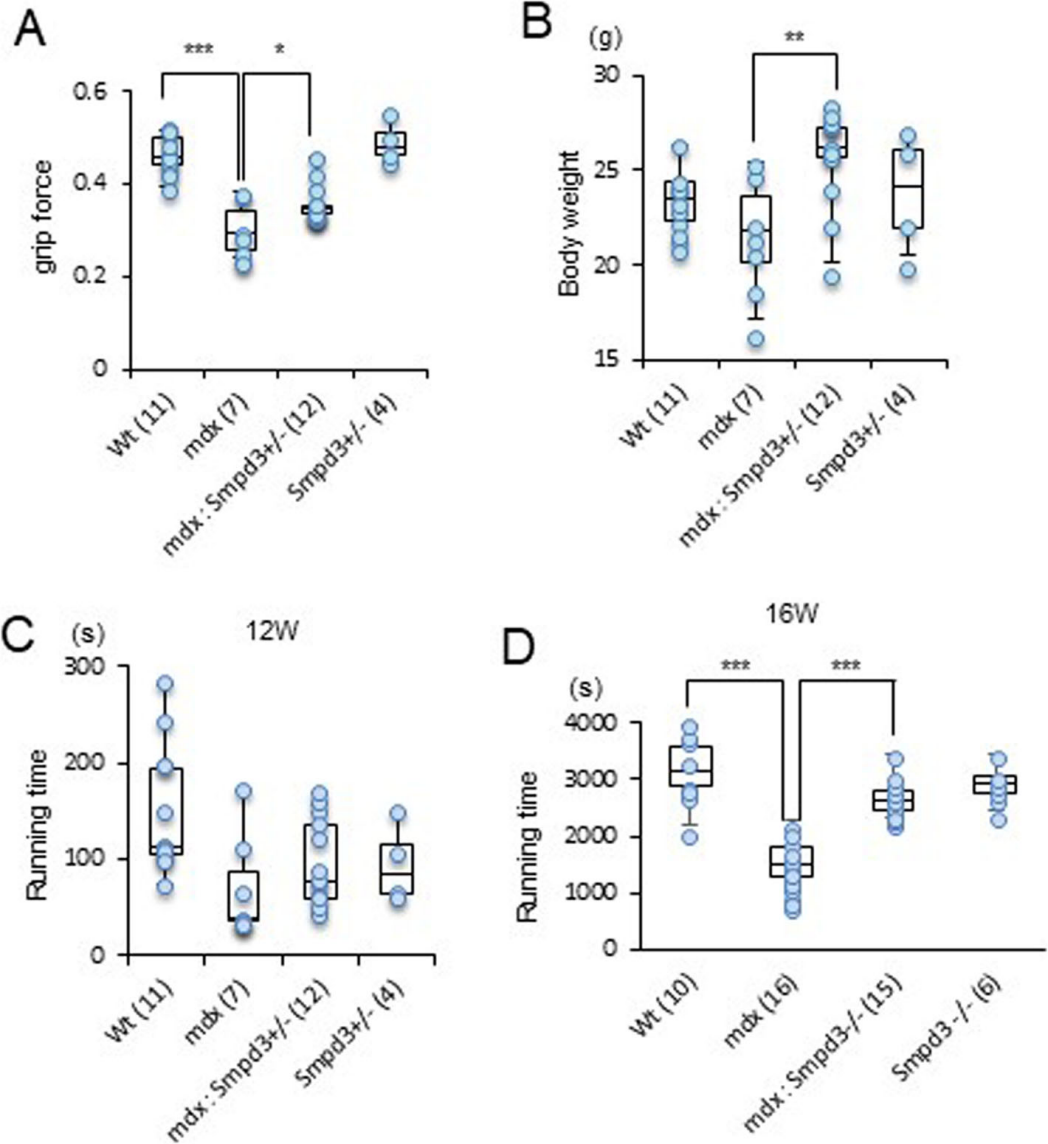
Ablation of the *nSMase2*/*Smpd3* gene in the *mdx* genetic background enhances muscle performance. Grip force (**a**), body weight (**b**), and average time spent running on the treadmill in wt, *mdx*, and *mdx:Smpd3*^*+/−*^ (#11: *n* = 3; #28: *n* = 9) mice at 12 weeks of age (**c**) and in wt, *mdx*, *mdx:Smpd3*^*−/−*^ (#238), and *Smpd3*^*−/−*^ (#29) mice at 16 weeks of age (**d**). The number of animals used is indicated in parentheses. *p < 0.05, **p < 0.01, ***p < 0.001

To further assess whether the reduction in muscle membrane permeability caused by *nSMase2/Smpd3* KO in *mdx* mice improved muscle performance, we assessed muscle force and endurance. The grip strength test demonstrated that at 12 weeks of age, the muscle strength of *mdx* mice was significantly lower than that of wt mice (*p* < 0.001), but that of *mdx:Smpd3*^*+/−*^ mice was significantly higher than that of *mdx* mice (Fig. 4a, *p* < 0.05). However, the *mdx:Smpd3*^*+/−*^ mice also weighed significantly more than the *mdx* mice (Fig. 4b, *p* < 0.01), and when grip strength was scaled to body weight, there was no difference between *mdx:Smpd3*^*+/−*^ and *mdx* mice (data not shown). An inclined treadmill running test indicated that *mdx:Smpd3*^*+/−*^ mice tended to run further than *mdx* mice, but the difference was not statistically significant (Fig. 4c). However, *mdx:Smpd3*^*−/−*^ mice ran for significantly longer than *mdx* mice at 16 (Fig. 4d, *p* < 0.001) and 60 (Additional file 15: Fig. S8, *p* < 0.05) weeks of age. These data show that loss of the *nSMase2/Smpd3* gene may improve dystrophic muscle function in *mdx* mice.

### *nSMase2/Smpd3* deletion modulates fiber size in dystrophic muscles of *mdx* mice

**Fig. 5:**
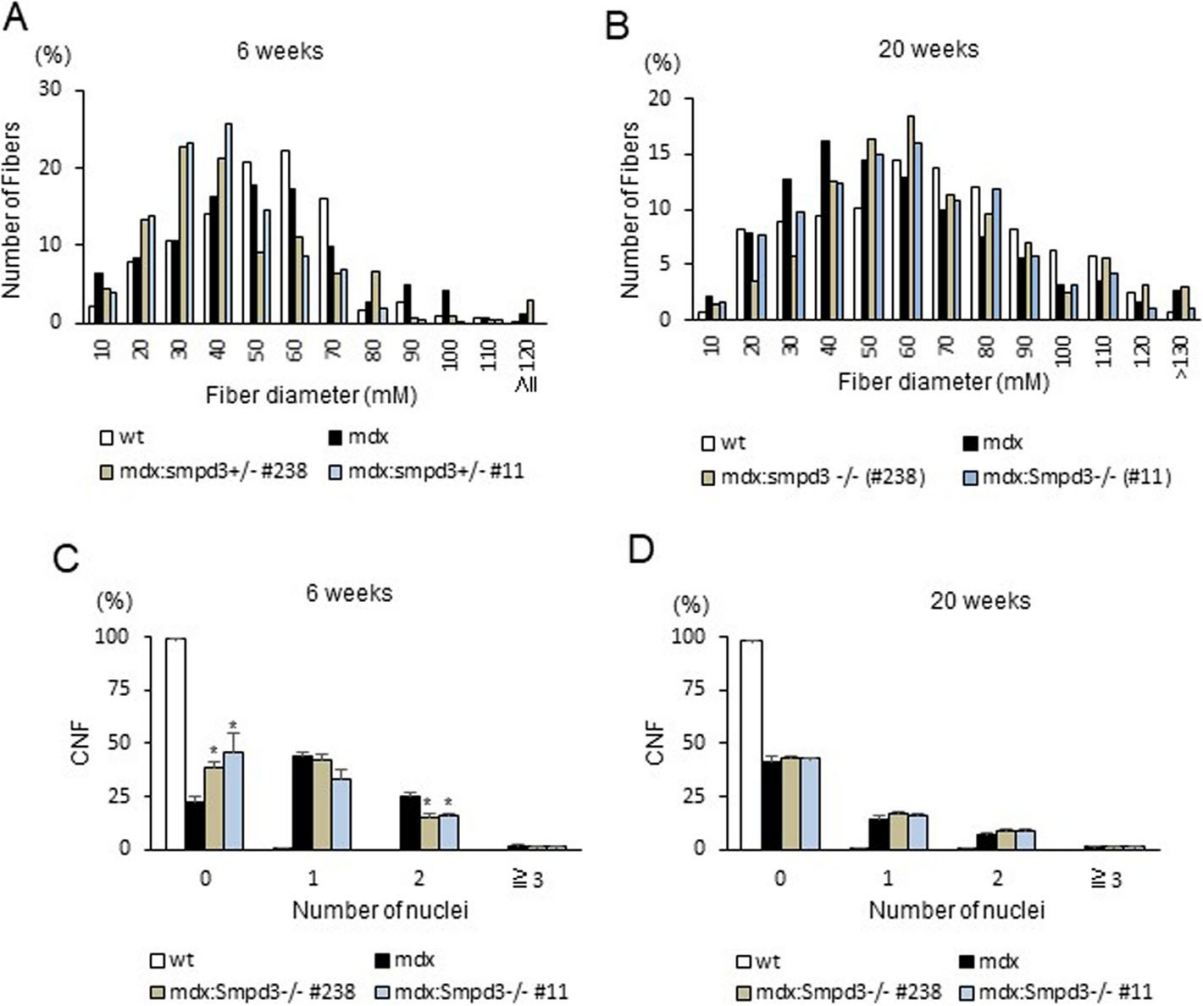
nSMase2/Smpd3 regulates fiber size and fusion in vivo. **a**, **b** Fiber size distribution in wt, *mdx*, and *mdx:Smpd3*^*−/−*^ (#11 and #238) mice at 6 (**a**) and 20 (**b**) weeks of age. **c**, **d** The proportion of fibers containing the indicated number of nuclei per fiber in wt, *mdx*, and, *mdx:Smpd3*^*+/−*^ (#11 and #238) mice at 6 weeks of age (**c**) and wt, *mdx*, and *mdx:Smpd3*^*−/−*^ (#11 and #238) mice at 20 weeks of age (**d**)

We analyzed muscle fiber diameter and central nucleation in wt, *mdx*, and *mdx:Smpd3*^*+/−*^ (#238 and #11) mice at 6 and 20 weeks of age. The muscle fibers were narrower in both lines of *mdx:Smpd3*^*+/−*^ mice than in *mdx* mice at 6 weeks (Fig. 5a), but were thicker at 20 weeks (Fig. 5b). At 6 weeks, there were significantly fewer centrally nucleated fibers (CNFs) containing two nuclei per fiber in both lines of *mdx:Smpd3*^*−/−*^ mice than in *mdx* mice (Fig. 5c, *p* < 0.05), but significantly more without central nuclei (Fig. 5c, *p* < 0.05). However, at 20 weeks there were no significant differences in the numbers of CNFs (Fig. 5d). These findings suggest that the loss of the *nSMase2/Smpd3* gene may affect fiber size via fiber fusion.

### Effects of the *nSMase2/Smpd3* gene on the survival, proliferation, and differentiation of myogenic cells in *mdx* mice

Next, we hypothesized that *nSMase2/Smpd3* regulates muscle regeneration via the survival and apoptosis of dystrophic myofibers. To test this hypothesis, we measured caspase-3 and caspase-9 activity in the SM and cerebellum of wt, *mdx*, and *mdx:Smpd3*^*−/−*^ (#238) mice. At 12 weeks, caspase-3 activity in the GAS and TA muscles of *mdx:Smpd3*^*−/−*^ mice was significantly lower than in *mdx* mice (Additional file 16: Fig. S9A, *p* < 0.05), but there were no differences between them for the GAS muscle at 9 or 18 weeks or for the cerebellum at 18 weeks (Additional file 16: Fig. S9A). There were also no differences between these lines in terms of caspase-9 activity in the GAS at either 9 or 12 weeks of age (Additional file 16: Fig. S9A). The expression levels of Bdnf protein in the GAS muscles of *mdx:Smpd3*^*−/−*^ mice at 5 (ns), 9 (*p* < 0.0.5), 12 (ns), and 14 (*p* < 0.05) weeks of age were lower than in *mdx* mice (Additional file 16: Fig. S9B–E), but there was no difference at 18 weeks (Additional file 16: Fig. S9F). We also performed a western blot analysis using an anti-caveolin-3 antibody as a lipid raft marker protein. At 14 weeks, caveolin-3 expression in the GAS muscle of *mdx* mice was significantly higher than in both wt and *mdx:Smpd3*^*−/−*^ mice (Additional file 16: Fig. S9E, *p* < 0.05).

To further investigate the effects of the loss of *nSMase2/Smpd3* in the degenerative muscles of *mdx* mice, we analyzed expression of various genes in the GAS muscles of wt, *mdx*, and *mdx:Smpd3*^*+/−*^ mice. The expression levels of *Pax7* at 14 weeks (*p* < 0.05) and *MuRF1* at 12 weeks (*p* < 0.001) in *mdx:Smpd3*^*+/−*^ mice were significantly lower than in *mdx* mice (Additional file 16: Fig. S9G). At 9 weeks, dysferlin C2A (Dysf_C2A) and integrin alpha5 (Itga5) in *mdx:Smpd3*^*−/−*^ (#238) mice were significantly upregulated, approximately 6-fold and 2-fold, respectively, relative to *mdx* mice (Additional file 16: Fig. S9H, *p* < 0.01), but at 12 and 19 weeks, these differences had disappeared (Additional file 16: Fig. S9H). At 19 weeks, *Tead1* expression in the GAS of *mdx:Smpd3*^*−/−*^ mice was significantly downregulated relative to *mdx* mice (Additional file 16: Fig. S9H, *p* < 0.05). On the other hand, at 12 weeks, *Igf-I* expression in the GAS of *mdx:Smpd3*^*−/−*^ (#11) mice was significantly lower (*p* < 0.05) than in *mdx* mice, but in line #238, it was somewhat higher (ns; Additional file 16: Fig. S9H). At 18 weeks, *mdx:Smpd3*^*−/−*^ mice exhibited significantly lower *Gata-2* expression than *mdx* mice (Additional file 16: Fig. S9H, *p* < 0.05). The expression of *Hsp72* in *mdx:Smpd3*^*−/−*^ (#238) mice was significantly upregulated relative to *mdx* mice (Additional file 16: Fig. S9H, *p* < 0.05), but not in line #11 mice (Additional file 16: Fig. S9H). In addition, at 12 weeks, the expression of *Has2* in the GAS of *mdx:Smpd3*^*−/−*^ (#238) mice was significantly upregulated relative to *mdx* mice (Additional file 16: Fig. S9H, *p* < 0.05).

The expression of the sarcolipin (*Sln*) gene, which encodes the *SERCA*-inhibitory peptide, in *mdx:Smpd3*^*−/−*^ (#238) mice was lower than in *mdx* mice at 9 weeks but higher at 14 weeks (Additional file 16: Fig. S9H). At 12 weeks, *mdx:Smpd3*^*−/−*^ mice exhibited significantly lower expression of the proapoptotic protein Bax than *mdx* mice (Additional file 16: Fig. S9H, *p* < 0.05). At 9 weeks, *mdx:Smpd3*^*−/−*^ (#238) mice exhibited significantly higher *cyclin D1* expression than *mdx* mice (*p* < 0.01), whereas at 12 (*p* < 0.01) and 19 (*p* < 0.05) weeks the *cyclin D1* expression levels in both #238 and #11 mice were substantially lower than in *mdx* mice (Additional file 16: Fig. S9H).

At 14 weeks of age, expression of the myogenic differentiation marker embryonic myosin heavy chain (*eMHC*)/myosin, heavy polypeptide 3 (*Myh3*) was significantly higher in the GAS of *mdx:Smpd3*^*−/−*^ (#238) mice than in *mdx* mice (*p* < 0.05), but not in #11 mice, although their expression level was significantly lower at 19 weeks (Additional file 16: Fig. S9H, *p* < 0.05). In addition, at 9 weeks, expression of myogenin (MyoG), another myogenic differentiation marker, in *mdx:Smpd3*^*−/−*^ (#238) mice was significantly higher than in *mdx* mice (*p* < 0.05), whereas at 19 weeks it was significantly lower (Additional file 16: Fig. S9H, *p* < 0.01).

At 9 weeks, *Mef2d* expression in the GAS of *mdx:Smpd3*^*−/−*^ mice was significantly higher than in *mdx* mice (*p* < 0.05), but at 19 weeks, their expression of *Mef2c* was significantly lower than that of *mdx* mice (Additional file 16: Fig. S9H, *p* < 0.05). At 14 weeks, the expression of *Pax7* in *mdx:Smpd3*^*−/−*^ (#238) mice was significantly lower than in *mdx* mice (Additional file 16: Fig. S9H, *p* < 0.05). At 12 weeks of age, the mitochondrial marker *Tfam* was significantly upregulated in *mdx:Smpd3*^*−/−*^ (#238) mice relative to *mdx* mice, but the reverse was true at 19 weeks (Additional file 16: Fig. S9H, *p* < 0.05). Expression of *myoglobin* at 9 and 12 weeks of age was higher in *mdx:Smpd3*^*−/−*^ (#238) mice than in *mdx* mice (Additional file 16: Fig. S9H, *p* < 0.05).

The expression of *Myf5* and *CD34*, the earliest markers of myogenic commitment, was slightly higher in *mdx:Smpd3*^*−/−*^ (#238 and #11) mice at 14 weeks of age than in *mdx* mice (Additional file 16: Fig. S9H). To test the specification of muscle fiber types, the expression of myosin heavy chain (*MyHC*) isoforms (types I, IIa, IIb, and IIx) were quantified using real-time PCR. At 12 weeks, expression of slow/oxidative type I and fast/oxidative type IIa fibers were unaffected by the loss of *nSMase2/Smpd3* in the *mdx* genetic background, but that of fast/glycolytic type IIb and fast/intermediate type IIx was higher in *mdx:Smpd3*^*−/−*^ mice than in *mdx* mice (Additional file 16: Fig. S9H). In 9-week-old *mdx:Smpd3*^*−/−*^ mice, levels of *Pgc-1alpha* and *troponin I (Tnnil1*) were significantly lower than in *mdx* mice (Additional file 16: Fig. S9H, *p* < 0.05).

The atrophy-associated genes *Foxo1*, *Foxo3*, *MuRF*, and *Gdf8* were also upregulated in *mdx:Smpd3*^*−/−*^ (#238) mice relative to *mdx* mice (Additional file 16: Fig. S9H). However, at 12 weeks, expression of the *Klf15* gene was significantly lower in *mdx:Smpd3*^*−/−*^ (#11) mice than in *mdx* mice (Additional file 16: Fig. S9H, *p* < 0.001). Expression of the *Hgf* gene in the GAS of *mdx:Smpd3*^*−/−*^ (#238) mice was higher than in *mdx* mice, but that of *Jag1*, *Notch1*, and *Notch3* was lower (Additional file 16: Fig. S9H).

Combined, these findings suggest that *nSMase2/Smpd3* knockdown might induce the regeneration of dystrophic muscle early in life, but that the opposite effect might be induced later on.

### Effects of expression of muscle-specific miRNAs on muscle health

It was recently reported that treatment of 10 DMD patients with an antisense oligonucleotide (NS-065/NCNP-01) that induces skipping of exon 53 in mutated dystrophin transcripts increased the dystrophin/spectrin ratio [34]. Using the sera of 10 DMD patients who had been administered with intravenous NS-065/NCNP-01 for 12 weeks, we quantified their expression of a set of muscle-abundant miRNAs (myomiRs) before and after this treatment. The median expression levels of miR-1, miR-133a, and miR-206 in all 10 patients after treatment were approximately 25%, 67%, and 20% lower than before treatment, respectively, but these results were not significant; only that of miR-133a approached significance (*p* = 0.08; Additional file 17: Fig. S10A–C).

We then divided the 10 patients into three groups according to the dose of NS-065/NCNP-01 they received (1.25, 5, or 20 mg/kg weekly). We compared the fold-change in the miRNA levels before (“pre”) and after (“post”) treatment and calculated these as log_2_[post/pre]. At a weekly dose of 5 mg/kg, the levels of all three miRNAs after treatment were lower than before treatment, and levels of miR-133a were reduced after treatment in all three groups (Additional file 17: Fig. S10D–F).

It has been shown that recovery of the dystrophin protein via the skipping of exon 23 in the mature dystrophin transcript normalizes serum myomiR levels [35, 36]. Given that NS-06/NCNP-01 induces the skipping of exon 53 in the dystrophin transcript and increases dystrophin protein expression, our results suggest that the restoration of extracellular myomiR levels is associated with the pathology of dystrophic muscles. To assess the association of serum extracellular vesicle (EV) content with muscular damage, we analyzed serum EV content in patients with seven different types of muscular disorder, and found that it was substantially lower in DMD patients than in Becker muscular dystrophy (BMD) patients (Additional file 17: Fig. S10G).

We then investigated these effects in our mouse lines. To assess whether deletion of the *nSMase2/Smpd3* gene in *mdx* mice restores myomiR levels, we quantified serum miR-1, miR-133a, and miR-206 levels in *mdx:Smpd3*^*+/−*^, *mdx:Smpd3*^*−/−*^, and *mdx* mice. Serum miR-1 and miR-206 levels (Additional file 17: Fig. S10H) and exosomal miR-133a levels (Additional file 17: Fig. S10I) in *mdx:Smpd3*^*+/−*^ mice were lower than in *mdx* mice. This was not the case for *mdx:Smpd3*^*−/−*^ mice at 9 and 14 weeks of age (Additional file 17: Fig. S10J–L), but their serum miR-133a levels were significantly higher than those of *mdx* mice at 19 weeks (Additional file 17: Fig. S10L). However, at 9, 14, and 19 weeks, serum levels of the muscular regeneration marker miR-31 in *mdx:Smpd3*^*−/−*^ mice were significantly higher than in *mdx* mice (Additional file 17: Fig. S10M).

Next, to analyze whether loss of the *nSMase2/Smpd3* gene in the *mdx* background affects muscular dystrophy via muscle-abundant miRNAs, we quantified the levels of the myomiRs miR-1, miR-133a, and miR-206 in the GAS muscles of *mdx* and *mdx:Smpd3*^*−/−*^ (#11 and #238) mice. The expression levels of miR-1 and miR-133a in *mdx* mice were lower than in wt mice, but that of miR-206 was higher (Additional file 18: Fig. S11). In contrast, levels of miR-1 and miR-133a were substantially higher in *mdx:Smpd3*^*−/−*^ (#238) mice than in *mdx* mice, while those of miR-206 were lower. *mdx:Smpd3*^*−/−*^ (#11) mice, on the other hand, exhibited no substantial differences in this respect from *mdx* mice (Additional file 18: Fig. S11).

We then investigated whether the benefits of the miRNA regulated by the *nSMase2/Smpd3* gene could lead to a recovery in the survival of myogenic cells. To address this issue, we incubated H2K cells in serum-depleted medium with or without the nSMase inhibitor GW4869 (0, 5, 10, 15, 20, 25, or 30 μM). Survival rates of myoblasts incubated for 24 h with 10–30 μM of GW4869 were significantly higher than in cells incubated without GW4869 (Additional file 19: Fig. S12A).

Next, to analyze the effects of myomiRs on cell survival, we assessed the survival of H2K myotubes that had or had not been transfected with myomiRs. The survival of H2K cells transfected with miR-1 was significantly poorer than that of cells without it, whereas significantly more of the H2K cells cultured with miR-206 survived than those without it (Additional file 19: Fig. S12B). We then assessed the effect of the nSMase2/Smpd3 protein on H2K cell survival under the overexpression of myomiRs by culturing H2K cells that had or had not been transfected with miR-1, miR-133a, or miR-206 with GW4869. The survival of those transfected with miR-133a was better than that of non-transfected cells (Additional file 19: Fig. S12C). In addition, the number of EVs in the supernatant of H2K cells was significantly higher than that in the supernatant of C_2_C_12_ cells (Additional file 19: Fig. S12D). The protein content of serum-derived EVs from *mdx:Smpd3*^*−/−*^ mice was significantly lower than that of *mdx* mice (Additional file 19: Fig. S12E and F).

### Loss of the *nSMase2/Smpd3* gene in *mdx* mice modulates anxiety behavior

**Fig. 6:**
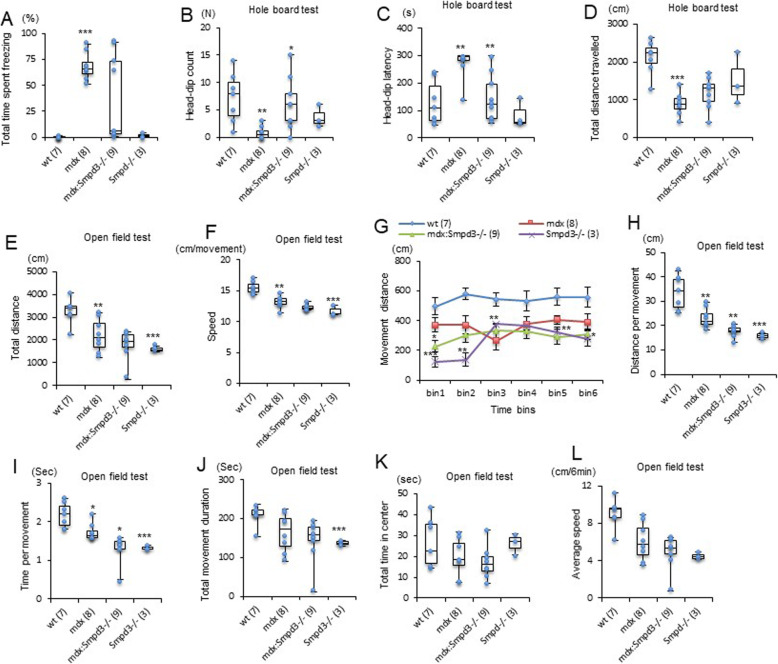
Loss of the *nSMase2*/*Smpd3* gene in *mdx* mice modulates anxiety-associated behavior in the hole-board and open-field tests. wt, *mdx*, and *mdx:Smpd3*^*−/−*^, and *Smpd3*^*−/−*^ mice at 11 weeks of age were restrained for 10 s and released into a measuring field. Freezing times were measured for 5 min. **a** The total time spent freezing during the 5 min period is indicated as a percentage. Head-dip count (**b**), head-dip latency (**c**), and the total distance traveled (**d**) in the hole-board test were analyzed in 12-week-old wt, *mdx*, *mdx:Smpd3*^*−/−*^, and *Smpd3*^*−/−*^ mice. Total distance (**e**), speed (**f**), movement distances in each of six time bins (**g**), distance per movement (**h**), time per movement (**i**), total movement duration (**j**), total time in the center (**k**), and average speed (**l**) were measured in the open-field test for wt, *mdx*, *mdx:Smpd3*^*−/−*^, and *Smpd3*^*−/−*^ mice at 10 weeks of age. The number of animals used is indicated in parentheses. *p < 0.05, **p < 0.01, ***p < 0.001

To investigate the effects of the *nSMase2/Smpd3* gene on emotional responses, *mdx* and *mdx:Smpd3*^*−/−*^ mice at 11–12 weeks of age were restrained for 10 s as an emotionally aversive stimulus. The mice were then released into a cage, and their behavior was monitored for 5 min for freezing vs locomotion. All the *mdx* mice froze for at least half of the 5-min period, whereas half of the *mdx:Smpd3*^*−/−*^ (#238) mice froze for much less than half of the time (Fig. 6a). Thus, the defense response was partially rescued in the *mdx:Smpd3*^*−/−*^ (#238) mice. We also investigated anxiety, emotionality, and the adaptive stress response to a novel environment using the hole-board test. The *mdx* mice performed significantly fewer head dips and exhibited significantly greater head-dip latency than both wt and *mdx:Smpd3*^*−/−*^ (#238) mice (Fig. 6b, c). The *mdx* mice also traveled a substantially shorter total distance than both of the other lines, although this difference was only significant in the comparison with the wt mice (Fig. 6d).

Next, to assess the role of the *nSMase2/Smpd3* gene in spontaneous behavior and reactivity in a novel environment, the open-field test was performed on 10-week-old wt, *mdx*, *mdx:Smpd3*^*−/−*^ (#238), and *Smpd3*^*−/−*^ (#238) mice. Although the *mdx* and *Smpd3*^*−/−*^ (#238) mice moved significantly more slowly and covered less distance than the wt mice, there was no significant difference between the *mdx* and *mdx:Smpd3*^*−/−*^ (#238) mice (Fig. 6e, f). However, in the first bin, the *mdx:Smpd3*^*−/−*^ (#238) mice covered substantially less distance than the *mdx* mice (Fig. 6g), and overall, they and the *Smpd3*^*−/−*^ mice covered significantly less distance and moved for significantly less time than the *mdx* mice (Fig. 6h, i). However, all three of these lines covered significantly shorter distances and spent less time in motion than wt mice (Fig. 6h, i). There were no significant differences in movement time, total time spent in the center of the field, or average speed among any of the four lines (Fig. 6j–l). Combined, these findings suggest that ablation of the *nSMase2/Smpd3* gene in *mdx* mice modulates their anxiety behavior and stress response.

### Roles of Bdnf, miRNA, and anxiety-associated genes in the abnormal behavior of *mdx* mice

To analyze whether *nSMase2/Smpd3* plays a role in the regulation of Bdnf expression in the hippocampus, we performed western blotting using an anti-Bdnf antibody on samples from *mdx* and *mdx:Smpd3*^*−/−*^ mice at various ages. There were no significant differences in Bdnf protein levels between *mdx* and *mdx:Smpd3*^*−/−*^ mice aged 5, 12, or 19 weeks (Additional file 20: Fig. S13A, C, and D). However, at 9 weeks, the Bdnf protein level in *mdx* mice was significantly lower than that of both wt and *mdx:Smpd3*^*−/−*^ mice (Additional file 20: Fig. S13B).

Next, to assess the effects of the nSMase2/Smpd3 protein on the transcription levels of the *Bdnf* isoforms in the hippocampus of *mdx* mice, we performed real-time qPCR using nine exon-specific primers in wt, *mdx*, and *mdx:Smpd3*^*−/−*^ mice aged 12 weeks (Additional file 20: Fig. S13E). In *mdx* mice, expression levels of five isoforms of *Bdnf*, exons I, II, V, VI, and VIII, were lower than in wt mice (Additional file 20: Fig. S13F), whereas in *mdx:Smpd3*^*−/−*^ mice, expression levels of all isoforms excluding exon IX were higher than in *mdx* mice (Additional file 20: Fig. S13G).

To assess myomiR-mediated regulation of *Bdnf* expression levels, we analyzed the transcriptional levels of myomiRs in the hippocampus. Although there were no significant differences in precursor-microRNA-1 levels between *mdx* and *mdx:Smpd3*^*−/−*^ mice at 5–19 weeks of age (Additional file 20: Fig. S13H), primary-microRNA-1 levels in *mdx:Smpd3*^*−/−*^ (#238 and #11) mice at 12 weeks were significantly lower than in *mdx* mice (Additional file 20: Fig. S13I). Although there were no significant differences in precursor-microRNA-133a levels between *mdx* and *mdx:Smpd3*^*−/−*^ mice at 12 weeks of age (Additional file 20: Fig. S13J), the precursor-microRNA-206 levels of *mdx:Smpd3*^*−/−*^ (#11) mice at this age were significantly higher than those of *mdx* mice (Additional file 20: Fig. S13K). To investigate the expression levels of genes induced in the hippocampus, we performed an expression analysis of 12-week-old wt, *mdx*, *mdx:Smpd3*^*+/−*^, and *mdx:Smpd3*^*−/−*^ (#238 and #11) mice using real-time qRT-PCR. The expression levels of dopamine receptor d1 (*Drd1*), cholecystokinin (*Cck*), the ionotropic glutamate receptor NMDA2B (*NR2b*), and postsynaptic density protein 95 (*PSD-95*) genes in *mdx:Smpd3*^*+/−*^ mice were significantly lower than in *mdx* mice (Additional file 20: Fig. S13L, *p* < 0.05). In addition, the expression levels of *Erg1* and *Arc* genes in *mdx:Smpd3*^*−/−*^ (#238) mice were slightly higher than those in *mdx* mice (Additional file 20: Fig. S13M). Expression levels of the dopamine receptor d2 *(Drd2)* and GIT ArfGAP 1 (*Git1*) genes were higher in *mdx:Smpd3*^*−/−*^ (lines #238 and #11, respectively) mice than in *mdx* mice (Additional file 20: Fig. S13M). These findings suggest that the loss of the *nSMase2/Smpd3* gene may regulate the anxiety phenotype of *mdx* mice via the regulation of *Bdnf* expression.

## Discussion

In this study, to investigate the role of the *nSMase2/Smpd3* gene in the dystrophic phenotypes of *mdx* mice, mutant mice with a deletion in the LIR of the nSMase2/Smpd3 protein were crossed with *mdx* mice to produce n*SMase2/Smpd3* dystrophin DKO mice. The DKO mice exhibited a reduced inflammation responses and less muscular degeneration in the early stages of the dystrophic process, but had exacerbated muscular necrosis in the later stages. We also found that the Bdnf pathway modulated the anxiety and stress responses of these DKO mice.

The nSMase/Smpd3 family of enzymes comprises four members: nSMase1, nSMase2, nSMase3, and mitochondria-associated nSMase. With the exception of nSMase3, these enzymes possess a DNase I-type catalytic core, suggesting a common mechanism for sphingomyelin catalysis [37]. Further, it has previously been shown that the mRNA expression level of *nSMase3* in brain is higher than that of *nSMase1* or *nSMase2* [38]. We observed a relatively large degree of variation in SMase activity in the hippocampi of line #11 *mdx:Smpd3*^*−/−*^ mice. This may have been due to the functional redundancy of other SMases.

We found less inflammation in the SM of *mdx:Smpd3*^*−/−*^ mice than in *mdx* mice. The acute phase of the pathology of *mdx* muscles (prior to four weeks of age) involves muscle inflammation with a bias toward M1 macrophages, which contributes to oxidative stress and muscle fiber lysis via the production of iNOS-derived NO, and promotes inflammation and myoblast proliferation via the production of Th1 cytokines [28, 39]. Later, at the age of 3 months, the arginase-expressing M2a macrophages that compete with iNOS for arginine can begin to reduce M1 macrophage cytotoxicity, and the muscles enter the regenerative phase.

The SM of dystrophin-deficient (*DMD*^*mdx*^) rats has been shown to be infiltrated by leukocytes, whose kinetics during the pathological course were parallel to those of serum CK levels in the SM, and the numbers of CD45^+^ mononuclear leukocytes at 4–16 weeks of age were significantly higher than in wt rats [27]. Of these increased muscle CD45^+^ mononuclear cells in *Dmd*^*mdx*^ rats, approximately 90% were CD68^+^ macrophages. In addition, the mononuclear cells from these rats expressed higher levels of transcripts for the cytokine TNF-alpha, which is associated with early muscle damage, than the same cells from wt rats. The inflammatory cytokines TNF-alpha, IL-6, and IL-1beta lead to increased endothelium permeability and promote early recruitment of innate immune cells, such as neutrophils and monocytes, which differentiate locally into inflammatory macrophages within injured tissues. Thus, leukocyte infiltration in the muscles of DMD model animals is associated with damaged muscle fibers and elevated serum CK levels.

In our study, expression levels of CD68, CD45, IL-6, and TNF-alpha in the SM of 12-week-old *mdx:Smpd3* DM mice were significantly lower than in *mdx* mice. This was also true for serum CK levels in 6–18-week-old *mdx:Smpd3* DM mice. These findings may suggest lower numbers of infiltrating mononuclear cells in *mdx:Smpd3* DM mice. In addition, the tissue recruitment of monocytes is mainly mediated by the chemokine Ccl2 and the chemokine receptor CCR2 or CCR5 via the blood circulation [40]. A deficiency in CCR2 in the SM of *mdx:CCR2* KO mice markedly and persistently reduced the infiltration of Ly6C^high^ inflammatory monocytes, which enter tissues in response to injury and differentiate into inflammatory M1 macrophages within inflamed tissues, relative to *mdx* controls, leading to a reduction in muscle fiber necrosis and endomysial fibrosis at 14 weeks of age [41]. On the other hand, in *mdx:CCR2* KO mice the abundance of intramuscular Ly6C^low^ macrophages, which patrol the vascular space with homing properties under steady-state conditions and comprise over 90% of resident macrophages, was significantly lower than in *mdx* controls during the early stages (4–9 weeks of age), but from 14 weeks to 6 months, it was similar to the *mdx* mice. The improvement in necrosis and fibrosis in the limb muscles of the *mdx:CCR2* KO mice was not sustained during the latter phase, possibly because of unsuppressed levels of profibrotic growth factors such as osteopontin.

It is likely that a mixture of monocyte-derived and tissue-resident macrophages accumulate in chronically inflamed tissues. However, the origins of the Ly6C^high^ and Ly6C^low^ monocytes/macrophages in dystrophic muscles are unclear. Although bone marrow has been thought to be the principal source of monocytes, it was recently reported that splenic Ly6C^high^ monocytes, which are outsourced from the bone marrow, contribute to recruitment and infiltration in dystrophic limb muscles, and to muscle fiber necrosis, during the early stages of the disease [42]. A reduction in infiltrated CD45^+^ cells in *mdx* mice improved muscle fiber necrosis and increased eMHC-positive regenerating fibers under a lack of splenic monocytes induced by splenectomy during the early phases of the condition. However, during the late stages, dystrophic muscle regeneration is impeded by reduced angiogenesis and increased fibrosis. This causes a delay in the phenotypic shift from proinflammatory to proregenerative macrophages, which affects the tissue cytokine environment. Thus, the optimal response to chronic tissue injury in dystrophic muscle depends on the fine balance between the phenotypes of macrophage types, and the cytokine environment may be critical for the progression of the pathology.

It has also been shown that nSMase2/Smpd3 deficiency or inhibition strongly suppresses M1 macrophage infiltration and differentiation, and inhibits inflammation, in a mouse model of atherosclerosis via Nrf2 (NF-E2-related factor 2)/HO-1 pathway activation. In this pathway, the rapid nuclear translocation and accumulation of Nrf2 protein was promoted to inhibit early cytokine response, such as IL-1beta and IL-6. This may occur via persistent Akt phosphorylation through the reduction of the ceramide-induced inhibition of PP2A (protein phosphatase 2) activity and the suppression of the expression of inflammatory and adhesion genes such as Ccl2 and ICAM-1 and/or ceramide [20, 43]. Given these reports, the beneficial effects in the early stages and adverse effects in later stages in our *mdx:Smpd3* DKO mice may have been caused partially by the inhibition of monocyte/macrophage recruitment and inflammatory responses. Furthermore, older *mdx:Smpd3* DKO mice might display more severe dystrophic phenotypes, such as necrosis and fibrosis, which could more clearly be distinguished from the effects seen in younger *mdx:Smpd3* DKO mice, such as the increased serum CK levels in 28-week-old *mdx:Smpd3* DKO (#238 and #11) mice and the increased number of EBD-positive fibers in 20-week-old *mdx:Smpd3* DKO (#11) mice.

Splenectomized *mdx* and *mdx:CCR2* DM mice do not show improvements in muscle strength at 14 weeks or 6 months [41, 42]. On the other hand, in our study, the running performance of the *mdx:Smpd3* DM mice at 16 and even 60 weeks in the treadmill exhaustion test was better than that of the *mdx* mice. Combined, this evidence suggests that suppression of the inflammation of the monocytes/macrophages may not contribute to the improvement in muscle performance in the nSMase2/Smpd3-deficient *mdx* mice.

In general, degenerative/necrotic lesions in the dystrophic muscles of *mdx* mice exist in small clusters, possibly due to an imbalanced tissue environment caused by the repeated degeneration–regeneration cycles, during which inflammatory cells such as monocytes and macrophages are recruited and infiltrate muscle cells. The pathologic changes in *mdx:Smpd3* mice during the early stages were spatiotemporally restricted to specific areas, suggesting that the main cause of the pathologic changes in the SM of *mdx:Smpd3* mice may be the inhibition of monocyte/macrophage recruitment into the dystrophic lesions. However, during the later stages, the dystrophic lesions in the SM of *mdx:Smpd3* mice were sparse but worsened despite the improvements in muscle performance. This indicates that the changes in the dystrophic pathology within their SM, such as susceptibility to exercise-induced injury, oxidative stress, and impaired regeneration capacity, may have been caused by multifactorial pathways. Possible candidates are decreased cytosolic and mitochondrial calcium concentrations, calpain inactivation [17], and inhibition of phosphorylation of the Stat1/Stat3 transcriptional factor, in addition to the sustained inhibition of Lyc6C^high^ monocyte/macrophage recruitment and suppressed anti-inflammatory differentiation. Additionally, the dual CCR2/CCR5 chemokine receptor antagonist has been shown to reduce macrophage infiltration and decrease the prevalence of regenerated CNFs, which are thought to be a marker of previous necrosis–regeneration events, in the diaphragm of *mdx* mice [44]. Thus, the significant reduction in CNFs containing multiple nuclei in the *mdx:Smpd3* DM mice may also have been caused by the inhibition of monocyte/macrophage infiltration.

The inhibition of TRPV2 (transient receptor potential cation channel), which is a principal Ca^2+^-entry route, leads to a sustained Ca^2+^ increase and muscle degeneration in two DMD mouse models [45]. Dystrophic pathologies, such as increased abundance of CNFs, variability in fiber size, increased Ca^2+^ levels in muscle fibers, elevated serum CK levels, and reduced muscle performance, are all ameliorated by the inhibition of TRPV2 in the early stages (4–10 weeks), when the degeneration–regeneration cycles are ongoing. However, in old mice (> 26 weeks), when the potential for such cycles may have been exhausted, the improvements in abundance of CNFs and fiber size variability are slight [45]. Also, since necrosis could play a critical role in mediating the myocyte and myofiber loss associated with calcium dysregulation, the inhibition of cyclophilin D which directly regulates the changes in mitochondrial permeability that depend on calcium and reactive oxygen species causes noticeable improvements in muscular dystrophies, such as reductions in CNFs, fibrosis, and myofiber necrosis [46].

Dystrophic phenotypes such as the increased abundance of CNFs, fibrosis, calpain activation, and serum CK levels that are seen in *mdx* mice at 6 weeks to 3 months have been shown to be improved by SM-specific overexpression of sarcoplasmic reticulum Ca^2+^ ATPase 1 (SERCA1). This enzyme reverses a defect in sarcoplasmic reticulum Ca^2+^ reuptake that causes dystrophic fibers and reduced total cytosolic Ca^2+^ [31]. In addition, reducing sarcolipin, which is an inhibitor of SERCA and is abnormally elevated in both the slow- and the fast-twitch SM of DMD patients and animal models, mitigates dystrophic phenotypes and improves muscle regeneration via the restoration of SERCA function [47]. Increased expression of intramuscular heat shock protein 72 (Hsp72) also ameliorates the dystrophic pathway and preserves muscle strength, via an interaction with SERCA that enhances its function [48]. On the other hand, nSMase2/Smpd3 deficiency suppresses the ceramide-dependent activation of protein phosphatase 2a (PP2a) that maintains Akt phosphorylation, thereby inducing hyaluronan synthase 2 (HAS2) and Hsp72 expression [49, 50]. The amelioration of dystrophic phenotypes in young *mdx:Smd3* DM mice may therefore contribute to protection from mitochondrial Ca^2+^-overload-induced myofiber necrosis.

It has also been shown that C5a, sICAM-I, IL-1ra, IL-16, Ccl2, TIMP-1, and TNF-alpha levels are higher in *mdx* mice than in wt mice [32]. The levels of all seven of these cytokines and chemokines in the GAS muscle of the *mdx:Smpd3*^*−/−*^ mice were lower than in the *mdx* mice, as were the expression levels of *TNF-alpha* and *IL-6*. A C5a inhibitor rescued the decreased force and increased the abundance of necrotic fibers in the *mdx* mice via a change to fast-twitch fibers, and also increased the maturation of macrophages [32]. A coordinated balance between pro-inflammatory and anti-inflammatory macrophages is important for successful muscle repair. However, in dystrophic muscle, this balance of inflammatory responses might be disrupted. Bdnf is produced by immune cells such as CD4^+^ and CD8^+^ T lymphocytes and monocytes/macrophages. It is modulated by TNF-alpha and IL-6, which are located near regenerating fibers that are positive for p75NTR, and CD56/NCAM owing to the repair of tissue in inflamed muscle [51, 52]. The *Bdnf–p75NTR* axis positively regulates the tissue-protection response [52].

The crosstalk between immune and muscle cells, such as macrophages and SCs, can positively regulate homeostasis, proliferation, and the repair of myogenic cells via chemokines and cytokines that originate from infiltrated monocytes/macrophages [52]. The macrophages play either a supportive or a deleterious role in cells via the amplification or downregulation, respectively, of inflammatory responses that promote the elimination of myogenic debris and prevent excessive tissue damage. The role they play depends on their activation state, which is affected by changes in their environment. However, their role in either the promotion or mitigation of the pathogenesis of dystrophy is unclear. In our study, Bdnf protein expression was higher in the GAS of *mdx* mice than in both wt and *mdx:Smpd3*^*−/−*^ mice at 9 and 14 weeks of age. Thus, the infiltration of circulating monocytes might be reduced in *mdx:Smpd3*^*−/−*^ mice.

It has been shown that repression of Bdnf synthesis depends on cell differentiation in the SM [53]. Bdnf was highly expressed in muscle Pax7-positive SCs and myoblasts in culture, whereas its expression was absent or very low in myofibers, and was repressed after myogenic differentiation. In addition, the overall levels of *Bdnf* mRNA are strongly correlated with those of a progenitor marker, *Pax3*, in mature muscles. By reducing endogenous levels of Bdnf, myoblasts engage in early myogenic differentiation, despite the presence of growth media. This evidence indicates that the primary role of Bdnf in SM is to maintain the population of SCs by preventing their myogenic differentiation.

The expression levels of the *MyoG* and *eMHC*/*Myh3* genes in the GAS of the *mdx:Smpd3*^*−/−*^ mice were higher in the early stages and lower in the later stages than in the *mdx* mice. In addition, expression levels of the early myogenic markers *Myf5* and *CD34* were higher in the GAS of *mdx:Smpd3*^*−/−*^ mice than in *mdx* mice. However, *Pax7* expression in the GAS of *mdx:Smpd3*^*−/−*^ mice was significantly lower than in *mdx* mice. Also, it has been shown that *Pax7* expression is directly targeted by the SM-specific miR-431, which promotes myogenic differentiation via the upregulation of MyoG and mitochondrial transcription factor A (*Tfam*) [54]. However, the expression level of *Myf5* was not affected by miR-431. This suggests that *Pax7* expression in *mdx:Smpd3* DKO mice may be regulated by miR-431.

These results imply that the severity of the dystrophy in *mdx:Smpd3*^*−/−*^ mice may be ameliorated via modulation of the differentiation balance of SCs in dystrophic muscles. It has been shown that chronic overactivation of Notch signaling occurs in severely dystrophic muscles with impaired muscle regeneration [55]. In this study, *mdx:Smpd3*^*−/−*^ mice exhibited downregulation of the *Jag1*, *Notch1*, and *Notch3* genes relative to *mdx* mice. *Jag1*, induced by IL-1beta, suppresses the muscle regeneration capacity of DMD muscles, probably through *Notch3* activation [56]. This evidence suggests that *nSMase2/Smpd3* regulates muscle regeneration with SCs via the Notch and Pax7 pathways.

We also analyzed the defensive behavior of the *mdx:Smpd3*^*−/−*^ mice and found that the abnormal behavior exhibited by *mdx* mice was completely reversed in half of the DKO mice observed. In addition, the *mdx:Smpd3*^*−/−*^ mice performed significantly fewer head-dips (a marker of anxiety behavior in the hole-board test) than the *mdx* mice. In the CA1 region of the hippocampus of *mdx* mice, abnormal synaptic plasticity caused by a reduction in gamma aminobutyric acid (GABA) efficacy has been reported [14, 57]. *nSMase2/Smpd3* inhibition blocks TNF-alpha-induced excitatory postsynaptic currents from CA1 pyramidal cells [29]. In our study, Bdnf protein expression in the hippocampus of *mdx* mice was significantly lower than in wt mice, but loss of the *nSMase2/Smpd3* gene ameliorated this effect. Bdnf secretion promotes GABAergic synaptogenesis [58]. Synaptic GABA_A_-Rs are sensitive to benzodiazepines, drugs with robust anti-anxiety effects that bind exclusively to GABA_A_-Rs. They are predominantly located within non-lipid raft fractions and enhance the potentiating effects of benzodiazepines by impairing lipid raft integrity. This suggests that the localization of receptors in lipid rafts affects the potency and efficacy of neurotransmitter signaling and that this plays a role in neurological disorders [59, 60]. Thus, the association of lipid rafts with GABA_A_-R appears to be a downregulatory mechanism for selective synaptic transmission and plasticity. In addition, the disruption of lipid rafts blocks the potentiating inhibitory effects of Bdnf in GABA_A_-R signaling via Bdnf-induced recruitment of the TrkB receptor into neuronal lipid rafts, creating selective synaptic plasticity [61, 62].

## Conclusion

In summary, our study shows that ablation of the *nSMase2/Smpd3* gene in *mdx* mice ameliorates membrane instability in the sarcolemma, improves muscle force and performance, and reduces excess inflammation in the early stages. Furthermore, the abnormal stress response of *mdx* mice was modulated by the loss of the *nSMase2/Smpd3* gene, possibly via Bdnf signaling. These findings suggest that signaling pathways modulated by the nSMase2/Smpd3 protein through lipid rafts might be novel therapeutic targets for DMD, via the stage-specific regulation of the expression levels of this protein and transcript, for example through exosomal transfer.

## Supplementary information

**Additional file 1 : Table S1.** PCR primers for real-time PCR.

**Additional file 2 : Table S2.** Nucleotide sequences of guide RNA for the nSMase2/Smpd3 gene.

**Additional file 3 : Table S3.** Oligonucleotide sequences for genotyping the nSMase2/Smpd3 and the dystrophin genes.

**Additional file 4 : Table S4.** Muscular disease patients analyzed in this study.

**Additional file 5 : Table S5.** SMase activity, inflammation area from TA sections, live-animal optical imaging, serum CK levels, number of EBD-positive fibers, force in grip test, body weight, runing times in treadmil test, caspase-3 activity, caspase-9 activity, myomiRs levels in ASO-treated DMD patients, exosome levels in patients of muscular diseases., freezing times in restrain test, hole board test, and open field test.

**Additional file 6 : Table S6.** Sample sizes and statistical powers.

**Additional file 7 : Fig. S1.** Expression of the *SMase/Smpd* gene family during C_2_C_12_ differentiation, and generation of *nSMase2/Smpd3* KO mice in the *mdx* genetic background using the CRISPR-Cas9 system. (**A**) Expression of *aSMase*/*Smpd1*, *nSMase*/*Smpd2*, *nSMase2*/*Smpd3*, and *Myh1* in C_2_C_12_ myotubes differentiated from day 1 to day 6, measured by real-time RT-PCR, which were normalized to glyceraldehyde 3-phosphate dehydrogenase (*gapdh*) gene expression. (**B**) Expression of the nSMase2/Smpd3 and Bdnf proteins in C_2_C_12_ myotubes differentiated from day 1 to day 6, detected by western blotting. Heat shock protein 90 (Hsp90) and gapdh were included as loading controls. Expression levels of nSMase2/Smpd3 and Bdnf were normalized by the expression level of Gapdh at each timepoint, and arbitrary units [AU] represent the band intensities in the western blot (*n* = 2). (**C**) Top: Amino acid sequences of the wt nSMase2/Smpd3 proteins and the six deletion mutants generated by the CRISPR-Cas9 system (#11, #20, #26, #28, #30, and #238). Amino acid deletions and substitutions are indicated in red. Phosphoserine sites are shown in green. Numbers on the top line show the amino acid positions. The asterisk represents a stop codon. Bottom: The domain structure of the nSMase2/Smpd3 protein, consisting of two N-terminal hydrophobic segments (HS1 and HS2: 1–84), a cytoplasmic juxtamembrane region (JX: 85–118), two catalytic domains (CD1 and CD2: 119–175 and 340–651), and a large insertion region (175–340). Red triangles indicate the targets of the sgRNAs. (**D**) Nucleotide sequences of the genomic region of exon 3 of the *Smpd3* gene in wt (bottom) and six deletion and/or insertion mutant mice (#11, #20, #28, #30, and #238) derived from (upper) the CRISPR-Cas9 system. Red and blue reverse triangles show deletion or/and insertion start and end sites, respectively. The identity of each mutant mouse line is indicated by the number preceded by # in the upper left corner of each nucleotide sequence.

**Additional file 8 : Fig. S2.** Generation of *nSMase2/Smpd3* KO mice in the *mdx* genetic background using the CRISPR-Cas9 system. (**A**) Genotyping results of the dystrophin gene in wt and *mdx* mice using mutation-specific primers that can detect the point mutation in the dystrophin gene. (**B**) Levels of the dystrophin protein in the tibia anterior of wt and *mdx* mice, which were mated with *Smpd3*^*−/−*^ mice to produce *mdx:Smpd3*^*−/−*^ mice.

**Additional file 9 : Table S9.** Founder mice.

**Additional file 10 : Fig. S3.** (**A, B**) Levels of the nSMase2/Smpd3 protein and the unrelated protein Gapdh in the gastrocnemius muscle (GAS) (**A**) and cerebellum (**B**) of wt, *mdx*, and *mdx:Smpd3*^*−/−*^ mice detected by western blotting. (**C**) Levels of the nSMase2/Smpd3 protein and the unrelated protein Gapdh in the hippocampi of wt, *mdx*, and *mdx:Smpd3*^*−/−*^ mice at the indicated ages, detected by western blotting. (**D, E**) nSMase2/Smpd3 enzymatic activities from the skeletal muscle (SM) (**D**) and hippocampus (**E**) of wt, *mdx*, and *mdx:Smpd3*^*−/−*^ mice from the indicated mouse lines (*n* = 3). * *p* < 0.05.

**Additional file 11 : Fig. S4.** Total array images of the expression of cytokines and chemokines in gastrocnemius (GAS) muscle (**A**) and diaphragm (**B**) of 12-week-old *mdx* (upper) and *mdx:Smpd3*^*−/−*^ (lower) mice. (**C**) The corresponding positions of each molecule within the array.

**Additional file 12 : Fig. S5.** The expression of inflammation-related genes in the diaphragm of wt, *mdx*, and *mdx:Smpd3*^*+/−*^ mice at 12 weeks of age was measured via real-time RT-PCR (*n* = 3 per genotype). * *p* < 0.05, ** *p* < 0.01.

**Additional file 13 : Fig. S6.** Real-time PCR analysis of inflammation gene markers in the gastrocnemius (GAS) muscle of *mdx* and *mdx:Smpd3*^*−/−*^ (#11 and #238) mice at the indicated ages. The number of animals used is indicated in parentheses. * *p* < 0.05, ** *p* < 0.01.

**Additional file 14 : Fig. S7.** *Smpd3* ablation reduces sarcolemmal instability in the muscles of young *mdx* mice but exacerbates it in older mice. (**A–C**) Serum CK levels of wt, *mdx*, *mdx:Smpd3*^*+/−*^ *(#11*, *#20*, *#26*, *#28*, *and #35*), *mdx:Smpd3*^*−/−*^*(#30)*, *Smpd3*^*+/−*^ *(#11*, *#26*, *#28*, *and #35*), and *Smpd3*^*−/−*^ *(#11)* mice at 6 (**A**: #26), 8–10 (**B**: #20, 28, #35, and #11), and 18 (**C**: #30) weeks of age. The number of animals used is indicated in parentheses. * *p* < 0.05, ** *p* < 0.01, *** *p* < 0.001.

**Additional file 15 : Fig. S8.** Mice lacking the *nSMase2*/*Smpd3* gene in the *mdx* genetic background showed enhanced muscle performance. The average time spent running on the treadmill for wt, *mdx*, *mdx:Smpd3*^*−/−*^ (#238), and *Smpd3*^*−/−*^ (#29) mice at 60 weeks of age are shown. The number of animals used is indicated in parentheses. * *p* < 0.05, ** *p* < 0.01.

**Additional file 16 : Fig. S9.** Effects of loss of the *nSMase2/Smpd3* gene in *mdx* mice on the proliferation, differentiation, and survival of myogenic cells. (**A**) Caspase-3 activity in the gastrocnemius (GAS; blue circles) and tibialis anterior (TA; yellow circles) muscles and cerebellum (orange circles); and caspase-9 activity in the GAS muscle (blue triangles) were measured in wt, *mdx*, and *mdx:Smpd3*^*−/−*^ mice at the indicated ages. Representative western blot analysis (upper) and quantitation of Bdnf (**B**–**F**) and caveolin-3 proteins (**E**) (lower) in the gastrocnemius (GAS) muscle of *mdx* and *mdx:Smpd3*^*−/−*^ mice at 5 (**B**), 9 (**C**),12 (**D**), 14 (**E**), and 18 (**F**) weeks of age. (**G**) Expression in the GAS of wt, *mdx*, and *mdx:Smpd3*^*+/−*^ mice at 12 weeks of age was measured via real-time RT-PCR (*n* = 3 per genotype). (**H**) Expression analysis of myogenesis-related genes in the GAS muscle at the indicated ages of *mdx*, *mdx:Smpd3*^*−/−*^ (#238), and *mdx:Smpd3*^*−/−*^ (#11) mice using qRT-PCR.The number of animals used is indicated in parentheses. * *p* < 0.05, ** *p* < 0.01, *** *p* < 0.001.

**Additional file 17 : Fig. S10.** miRNA levels in the sera of muscular dystrophy patients and *mdx* mice as diagnostic markers. miR-1 (**A**), miR-133a (**B**), and miR-206 (**C**) levels in the serum of Duchenne muscular dystrophy (DMD) patients before (pre) and after (post) treatment with the antisense oligonucleotides NS-065/NCNP-01 that induce exon skipping to correct the frame-shift. Post-treatment expression levels expressed as fold-changes relative to pre-treatment expression levels (based on **A**–**C**) of miR-1 (**D**), miR-133a (**E**), and miR-206 (**F**) at doses of 1.25 mg/kg (cohort 1), 5 mg/kg (cohort 2), and 20 mg/kg (cohort 3) in patients administered weekly with NS-065/NCNP-01 for 12 weeks. Expression levels were normalized to those of miR-16. The number of patients analyzed is indicated in parentheses. Extracellular vesicles (EVs) were extracted from the sera of patients with seven types of muscle disorder (DMD, Becker muscular dystrophy [BMD], distal myopathy with rimmed vacuoles [DMRV], facioscapulohumeral muscular dystrophy [LGMD], and limb-girdle muscular dystrophy 2B [LGMD2B]) and quantified based on acetylcholinesterase (AChE) activity (**G**). Levels of miR-1, miR-133a, and miR-206 in the sera (**H**) and in EVs isolated from the sera (**I**) of *mdx* and *mdx:Smpd3*^*+/−*^ mice, with expression levels normalized to spiked-in cel-39. Levels of miR-1 (**J**), miR-206 (K), miR-133a (**L**), and miR-31 (**M**) in the sera of *mdx* and *mdx:Smpd3*^*−/−*^ mice, normalized to U6. The number of animals used is indicated in parentheses.

**Additional file 18 : Fig. S11.** miR-1, miR-133a, and miR-206 levels in the GAS of wt, *mdx*, *mdx:Smpd3*^*−/−*^ (#11 and #238) mice at 12 weeks of age were analyzed using real-time qPCR.

**Additional file 19 : Fig. S12.** (**A**) H2K myotubes differentiated for three days were incubated with or without GW4869 (5, 10, 15, 20, 25, and 30 μM) in serum-depleted medium for 24 h. (**B, C**) H2K myotubes differentiated for six days and transfected with miR-1, miR-133a, and miR-206 were incubated in serum-depleted medium with or without GW4869 (25 μM) for four days. (**D**) Extracellular vesicles (EVs) were extracted and quantified based on acetylcholinesterase (AChE) activity from H2K and C_2_C_12_ cells. Data presented are the mean + standard error (SE) of absorbance at 450 nm of CCK-8. Each independent experiment was repeated at least three times. (**E**) EV content of the sera of 12-week-old wt, *mdx*, and *mdx:Smpd3*^*+/−*^ mice. (**F**) EVs were extracted from the sera of wt (*n* = 6), *mdx* (*n* = 6), and *mdx:Smpd3*^*−/−*^ mice (*n* = 12) and quantified based on AChE activity. * *p* < 0.05, ** *p* < 0.01.

**Additional file 20 : Fig. S13.** Expression analysis in the hippocampus of *mdx:Smpd3*^*−/−*^ mice. (**A**) Representative western blot analysis (top) of whole hippocampal protein homogenates from wt, *mdx*, and *mdx:Smpd3*^*−/−*^ mice at 5 weeks (**A**), 9 weeks (**B**), 12 weeks (**C**), and 19 weeks (**D**) of age using anti-Bdnf and anti-Gapdh antibodies. The ratio of mBdnf/Gapdh is expressed as arbitrary units [AU] (bottom). (**E–G**) Expression of *Bdnf* isoforms in the hippocampi of wt, *mdx*, and *mdx:Smpd3*^*−/−*^ mice at 12 weeks of age based on real-time RT-PCR. (**E**) Exon/intron structure and alternative transcripts of the mouse *Bdnf* gene. Exons are indicated as boxes and introns are indicated as lines. Filled black regions in the boxes indicate the translated regions, in which ATG represents the translated start codon. The expression levels of each *Bdnf* isoform from exon I to exon IX are shown as log_2_ (fold-changes) in *mdx* mice relative to wt mice (**F**) and in *mdx:Smpd3*^*−/−*^ mice relative to *mdx* mice (**G**). The levels of four myomiRs, precursor-microRNA-1 (**H**), primary-microRNA-1 (**I**), precursor-microRNA-133a (**J**), and precursor-microRNA-206 (**K**), were analyzed in the hippocampi of *Smpd3*^*−/−*^ (#11 and #238) and *mdx* mice at 12 weeks of age. (**L**) Expression of genes induced in the hippocampi of wt, *mdx*, and *mdx:Smpd3*^*+/−*^ mice at 12 weeks of age was measured via real-time RT-PCR (*n* = 3 per genotype). * *p* < 0.05, ** *p* < 0.01. (**M**) Expression levels of the *Nfactc1*, *Gabar2*, *Egr1*, *Arc*, *Drd2*, and *Git1* genes in the hippocampi of *mdx:Smpd3*^*−/−*^ and *mdx* mice. * *p* < 0.05.

## Data Availability

Datasets generated for this study are included in the article and Supplementary Material, and all experimental data are available upon reasonable request.
