# Supplementary figures and images for: The nSMase2/Smpd3 gene modulates the severity of muscular dystrophy and the emotional stress response in mdx mice
Source: BMC Med. 2020 Nov 19;18:343. doi: 10.1186/s12916-020-01805-5 (PMC7677854; doi:10.1186/s12916-020-01805-5)

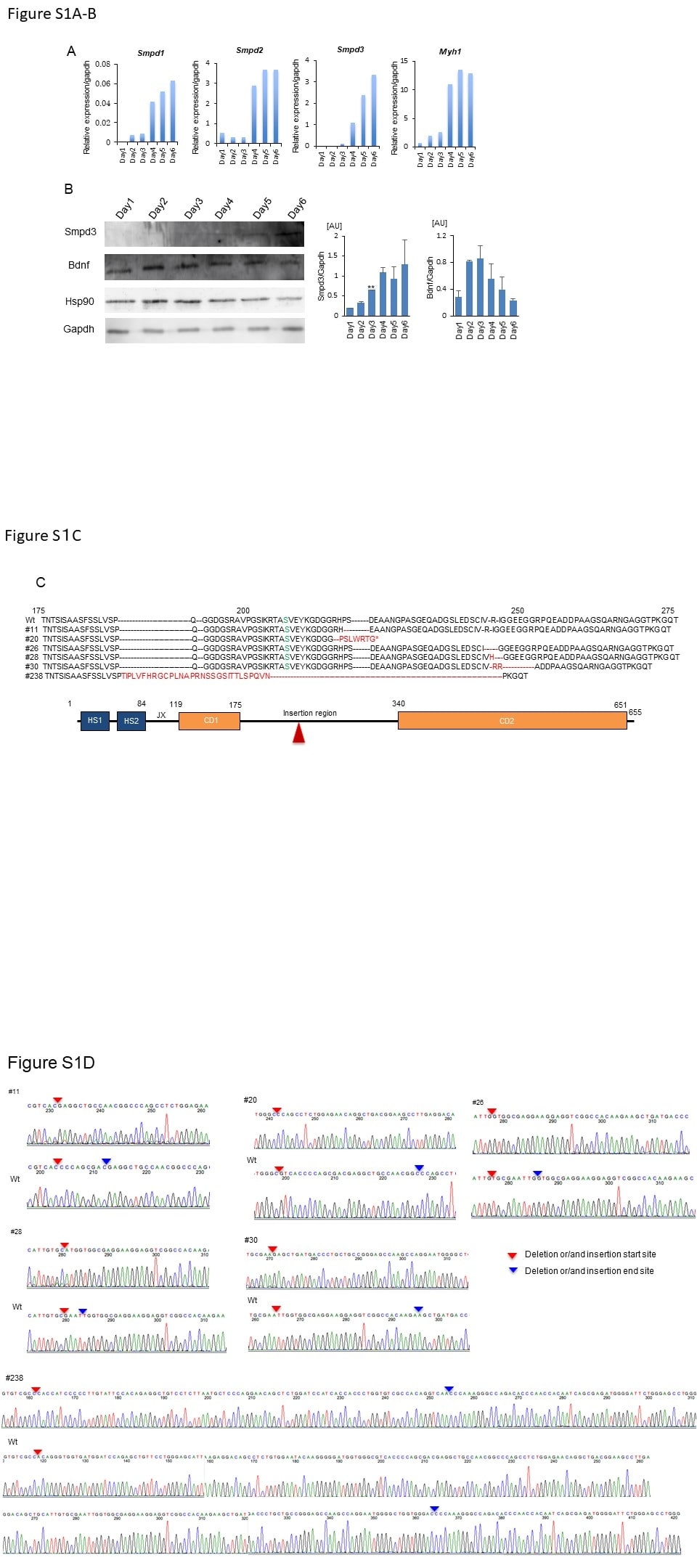

Supplement: Supplementary file 7 — Additional file 7 : Fig. S1. Expression of the SMase/Smpd gene family during C2C12 differentiation, and generation of nSMase2/Smpd3 KO mice in the mdx genetic background using the CRISPR-Cas9 system. (A) Expression of aSMase/Smpd1, nSMase/Smpd2, nSMase2/Smpd3, and Myh1 in C2C12 myotubes differentiated from day 1 to day 6, measured by real-time RT-PCR, which were normalized to glyceraldehyde 3-phosphate dehydrogenase (gapdh) gene expression. (B) Expression of the nSMase2/Smpd3 and Bdnf proteins in C2C12 myotubes differentiated from day 1 to day 6, detected by western blotting. Heat shock protein 90 (Hsp90) and gapdh were included as loading controls. Expression levels of nSMase2/Smpd3 and Bdnf were normalized by the expression level of Gapdh at each timepoint, and arbitrary units [AU] represent the band intensities in the western blot (n = 2). (C) Top: Amino acid sequences of the wt nSMase2/Smpd3 proteins and the six deletion mutants generated by the CRISPR-Cas9 system (#11, #20, #26, #28, #30, and #238). Amino acid deletions and substitutions are indicated in red. Phosphoserine sites are shown in green. Numbers on the top line show the amino acid positions. The asterisk represents a stop codon. Bottom: The domain structure of the nSMase2/Smpd3 protein, consisting of two N-terminal hydrophobic segments (HS1 and HS2: 1–84), a cytoplasmic juxtamembrane region (JX: 85–118), two catalytic domains (CD1 and CD2: 119–175 and 340–651), and a large insertion region (175–340). Red triangles indicate the targets of the sgRNAs. (D) Nucleotide sequences of the genomic region of exon 3 of the Smpd3 gene in wt (bottom) and six deletion and/or insertion mutant mice (#11, #20, #28, #30, and #238) derived from (upper) the CRISPR-Cas9 system. Red and blue reverse triangles show deletion or/and insertion start and end sites, respectively. The identity of each mutant mouse line is indicated by the number preceded by # in the upper left corner of each nucleotide sequence. [file 12916_2020_1805_MOESM7_ESM.jpg]

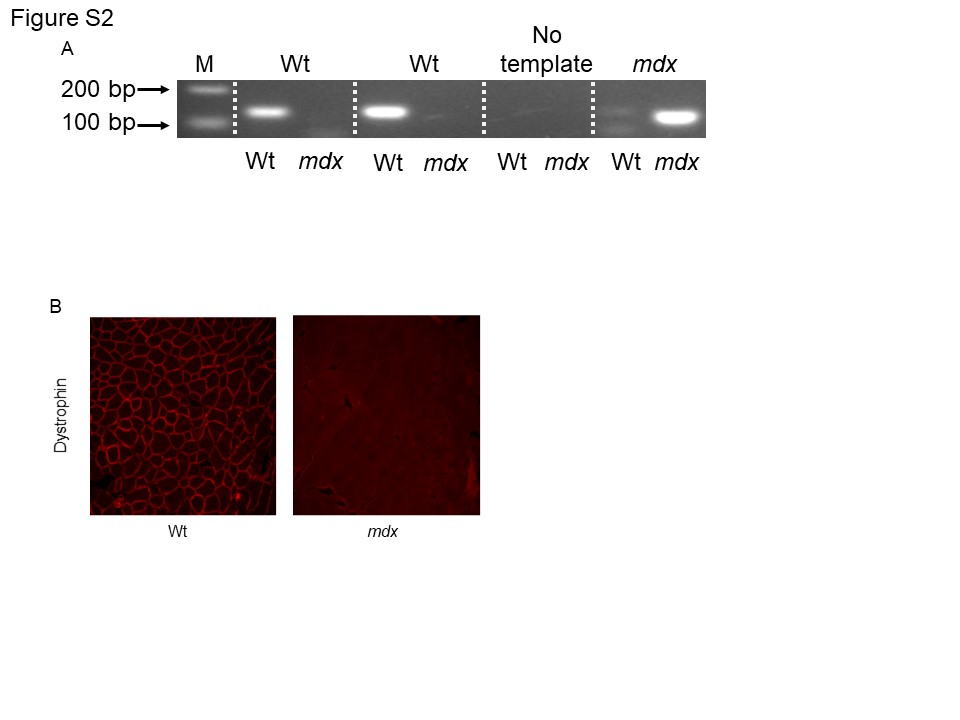

Supplement: Supplementary file 8 — Additional file 8 : Fig. S2. Generation of nSMase2/Smpd3 KO mice in the mdx genetic background using the CRISPR-Cas9 system. (A) Genotyping results of the dystrophin gene in wt and mdx mice using mutation-specific primers that can detect the point mutation in the dystrophin gene. (B) Levels of the dystrophin protein in the tibia anterior of wt and mdx mice, which were mated with Smpd3−/− mice to produce mdx:Smpd3−/− mice. [file 12916_2020_1805_MOESM8_ESM.jpg]

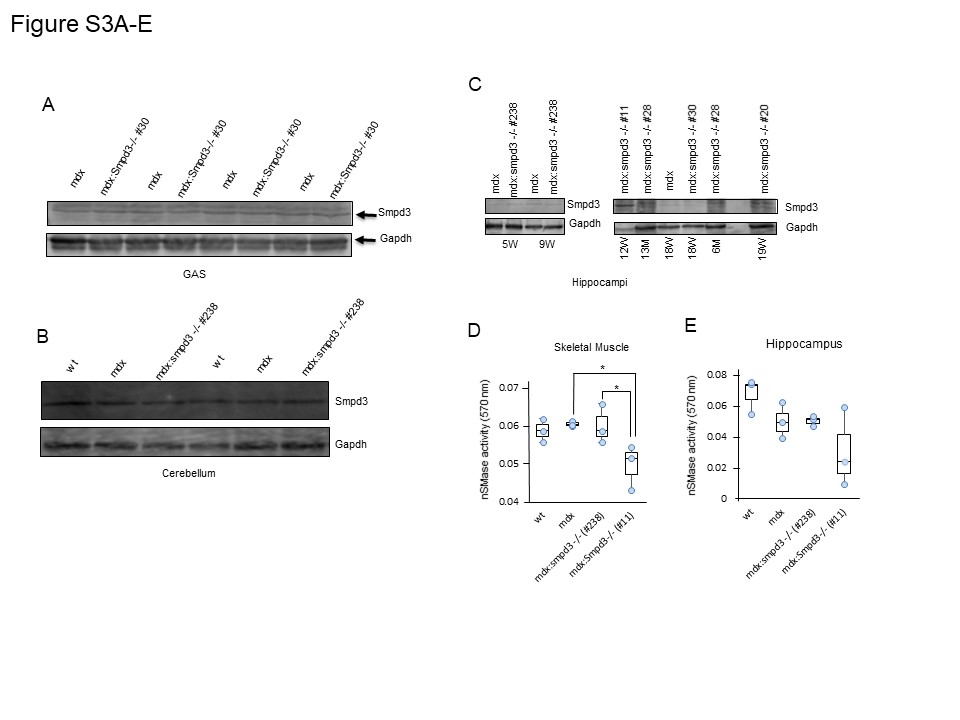

Supplement: Supplementary file 10 — Additional file 10 : Fig. S3. (A, B) Levels of the nSMase2/Smpd3 protein and the unrelated protein Gapdh in the gastrocnemius muscle (GAS) (A) and cerebellum (B) of wt, mdx, and mdx:Smpd3−/− mice detected by western blotting. (C) Levels of the nSMase2/Smpd3 protein and the unrelated protein Gapdh in the hippocampi of wt, mdx, and mdx:Smpd3−/− mice at the indicated ages, detected by western blotting. (D, E) nSMase2/Smpd3 enzymatic activities from the skeletal muscle (SM) (D) and hippocampus (E) of wt, mdx, and mdx:Smpd3−/− mice from the indicated mouse lines (n = 3). * p < 0.05. [file 12916_2020_1805_MOESM10_ESM.jpg]

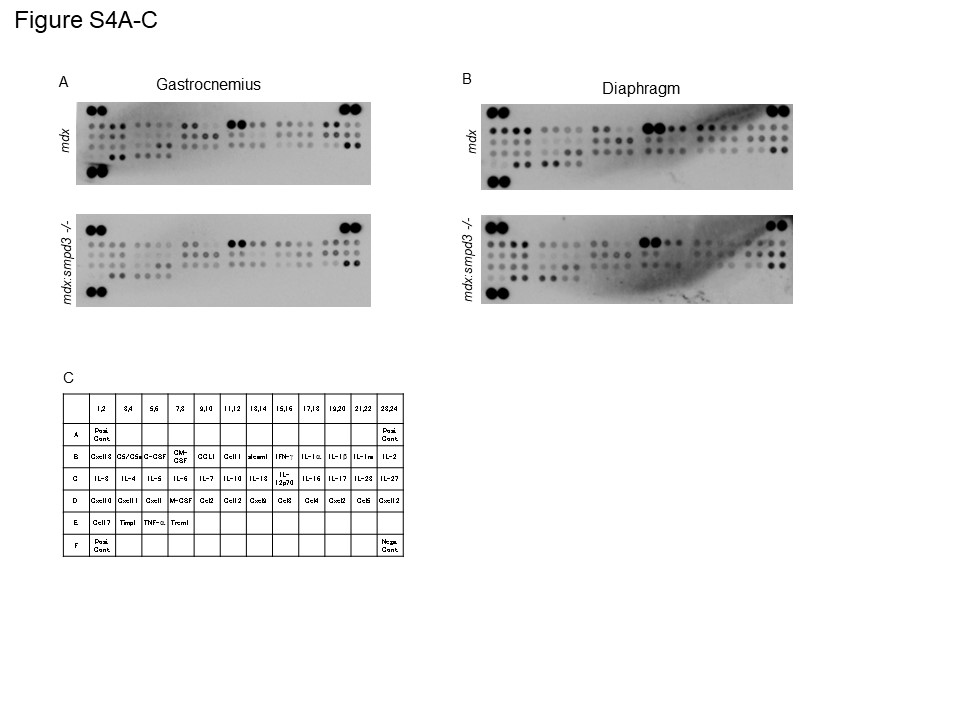

Supplement: Supplementary file 11 — Additional file 11 : Fig. S4. Total array images of the expression of cytokines and chemokines in gastrocnemius (GAS) muscle (A) and diaphragm (B) of 12-week-old mdx (upper) and mdx:Smpd3−/− (lower) mice. (C) The corresponding positions of each molecule within the array. [file 12916_2020_1805_MOESM11_ESM.jpg]

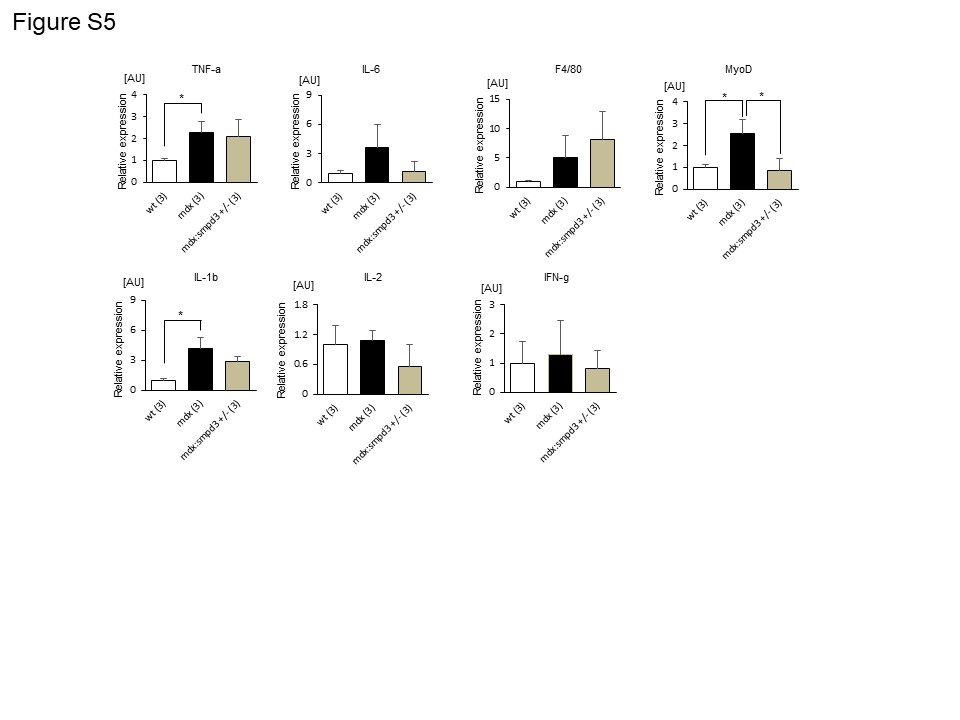

Supplement: Supplementary file 12 — Additional file 12 : Fig. S5. The expression of inflammation-related genes in the diaphragm of wt, mdx, and mdx:Smpd3+/− mice at 12 weeks of age was measured via real-time RT-PCR (n = 3 per genotype). * p < 0.05, ** p < 0.01. [file 12916_2020_1805_MOESM12_ESM.jpg]

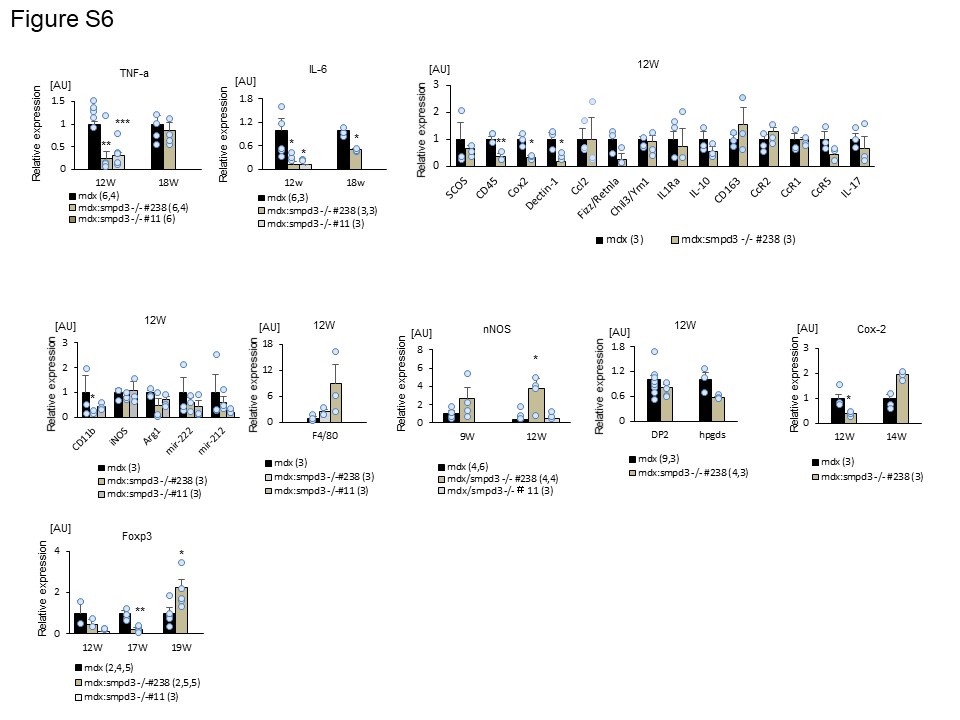

Supplement: Supplementary file 13 — Additional file 13 : Fig. S6. Real-time PCR analysis of inflammation gene markers in the gastrocnemius (GAS) muscle of mdx and mdx:Smpd3−/− (#11 and #238) mice at the indicated ages. The number of animals used is indicated in parentheses. * p < 0.05, ** p < 0.01. [file 12916_2020_1805_MOESM13_ESM.jpg]

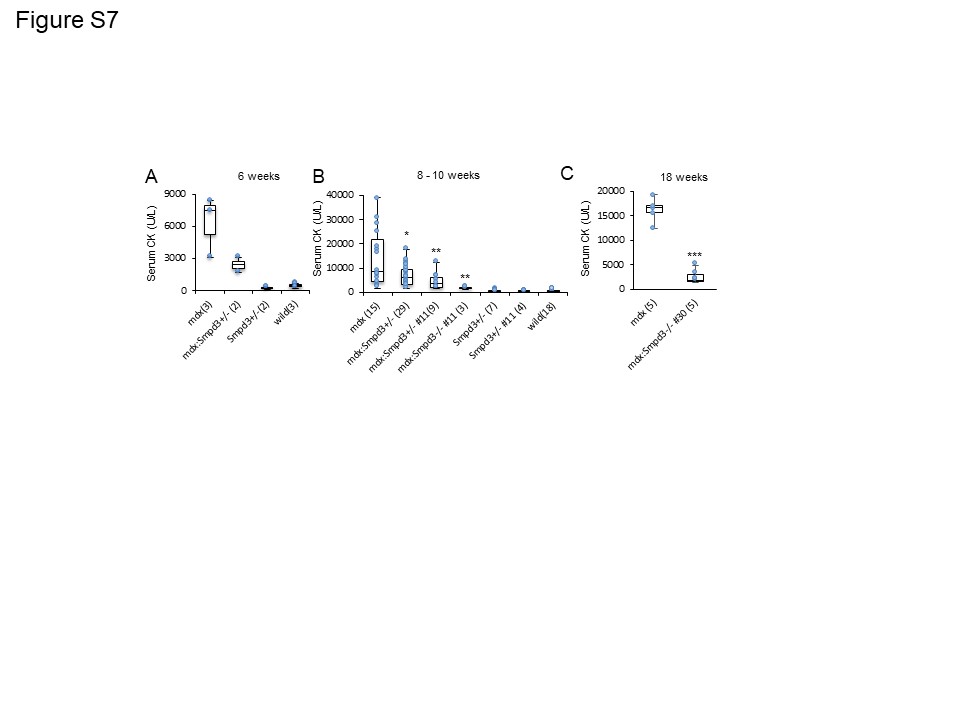

Supplement: Supplementary file 14 — Additional file 14 : Fig. S7. Smpd3 ablation reduces sarcolemmal instability in the muscles of young mdx mice but exacerbates it in older mice. (A–C) Serum CK levels of wt, mdx, mdx:Smpd3+/− (#11, #20, #26, #28, and #35), mdx:Smpd3−/−(#30), Smpd3+/− (#11, #26, #28, and #35), and Smpd3−/− (#11) mice at 6 (A: #26), 8–10 (B: #20, 28, #35, and #11), and 18 (C: #30) weeks of age. The number of animals used is indicated in parentheses. * p < 0.05, ** p < 0.01, *** p < 0.001. [file 12916_2020_1805_MOESM14_ESM.jpg]

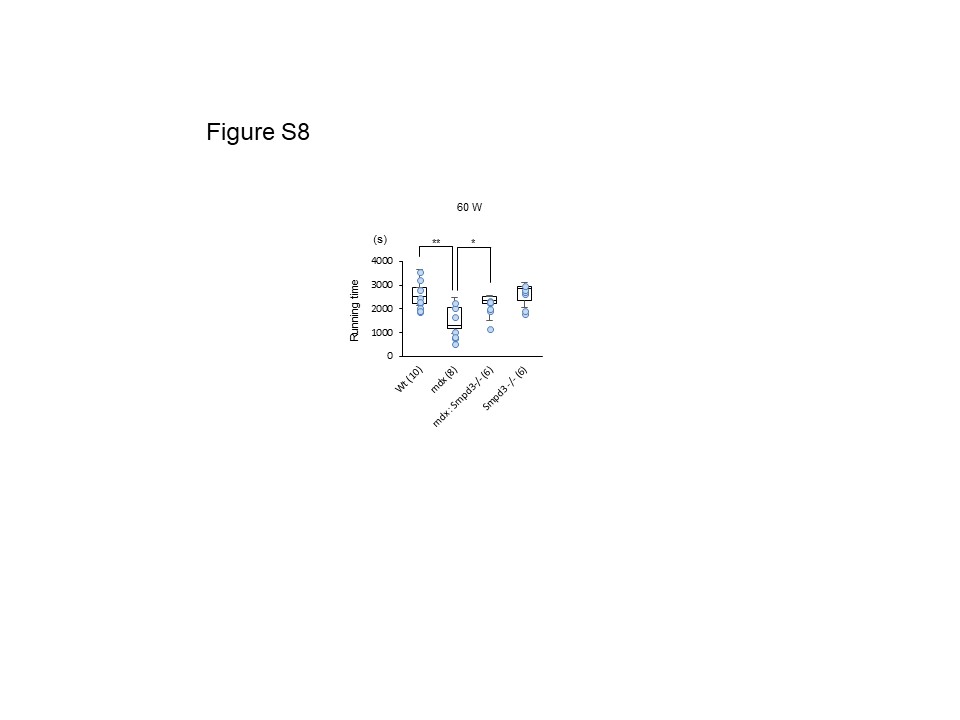

Supplement: Supplementary file 15 — Additional file 15 : Fig. S8. Mice lacking the nSMase2/Smpd3 gene in the mdx genetic background showed enhanced muscle performance. The average time spent running on the treadmill for wt, mdx, mdx:Smpd3−/− (#238), and Smpd3−/− (#29) mice at 60 weeks of age are shown. The number of animals used is indicated in parentheses. * p < 0.05, ** p < 0.01. [file 12916_2020_1805_MOESM15_ESM.jpg]

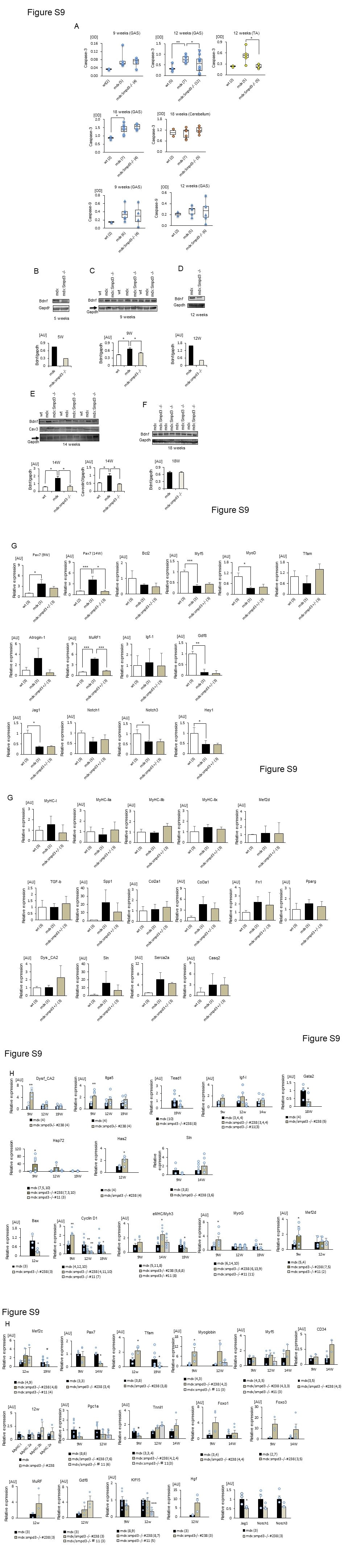

Supplement: Supplementary file 16 — Additional file 16 : Fig. S9. Effects of loss of the nSMase2/Smpd3 gene in mdx mice on the proliferation, differentiation, and survival of myogenic cells. (A) Caspase-3 activity in the gastrocnemius (GAS; blue circles) and tibialis anterior (TA; yellow circles) muscles and cerebellum (orange circles); and caspase-9 activity in the GAS muscle (blue triangles) were measured in wt, mdx, and mdx:Smpd3−/− mice at the indicated ages. Representative western blot analysis (upper) and quantitation of Bdnf (B–F) and caveolin-3 proteins (E) (lower) in the gastrocnemius (GAS) muscle of mdx and mdx:Smpd3−/− mice at 5 (B), 9 (C),12 (D), 14 (E), and 18 (F) weeks of age. (G) Expression in the GAS of wt, mdx, and mdx:Smpd3+/− mice at 12 weeks of age was measured via real-time RT-PCR (n = 3 per genotype). (H) Expression analysis of myogenesis-related genes in the GAS muscle at the indicated ages of mdx, mdx:Smpd3−/− (#238), and mdx:Smpd3−/− (#11) mice using qRT-PCR.The number of animals used is indicated in parentheses. * p < 0.05, ** p < 0.01, *** p < 0.001. [file 12916_2020_1805_MOESM16_ESM.jpg]

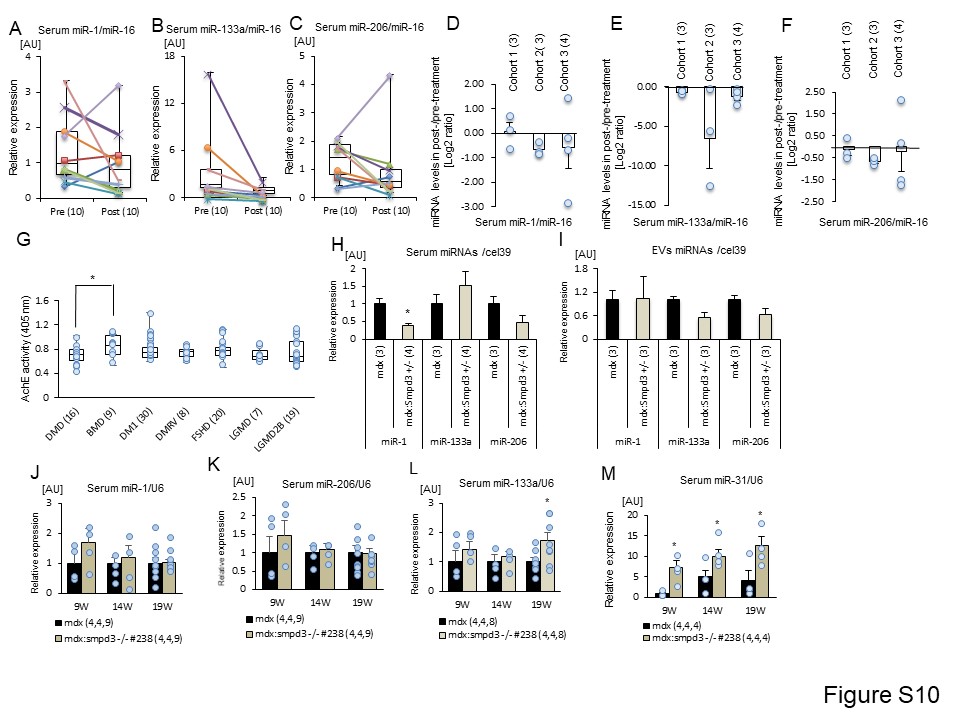

Supplement: Supplementary file 17 — Additional file 17 : Fig. S10. miRNA levels in the sera of muscular dystrophy patients and mdx mice as diagnostic markers. miR-1 (A), miR-133a (B), and miR-206 (C) levels in the serum of Duchenne muscular dystrophy (DMD) patients before (pre) and after (post) treatment with the antisense oligonucleotides NS-065/NCNP-01 that induce exon skipping to correct the frame-shift. Post-treatment expression levels expressed as fold-changes relative to pre-treatment expression levels (based on A–C) of miR-1 (D), miR-133a (E), and miR-206 (F) at doses of 1.25 mg/kg (cohort 1), 5 mg/kg (cohort 2), and 20 mg/kg (cohort 3) in patients administered weekly with NS-065/NCNP-01 for 12 weeks. Expression levels were normalized to those of miR-16. The number of patients analyzed is indicated in parentheses. Extracellular vesicles (EVs) were extracted from the sera of patients with seven types of muscle disorder (DMD, Becker muscular dystrophy [BMD], distal myopathy with rimmed vacuoles [DMRV], facioscapulohumeral muscular dystrophy [LGMD], and limb-girdle muscular dystrophy 2B [LGMD2B]) and quantified based on acetylcholinesterase (AChE) activity (G). Levels of miR-1, miR-133a, and miR-206 in the sera (H) and in EVs isolated from the sera (I) of mdx and mdx:Smpd3+/− mice, with expression levels normalized to spiked-in cel-39. Levels of miR-1 (J), miR-206 (K), miR-133a (L), and miR-31 (M) in the sera of mdx and mdx:Smpd3−/− mice, normalized to U6. The number of animals used is indicated in parentheses. [file 12916_2020_1805_MOESM17_ESM.jpg]

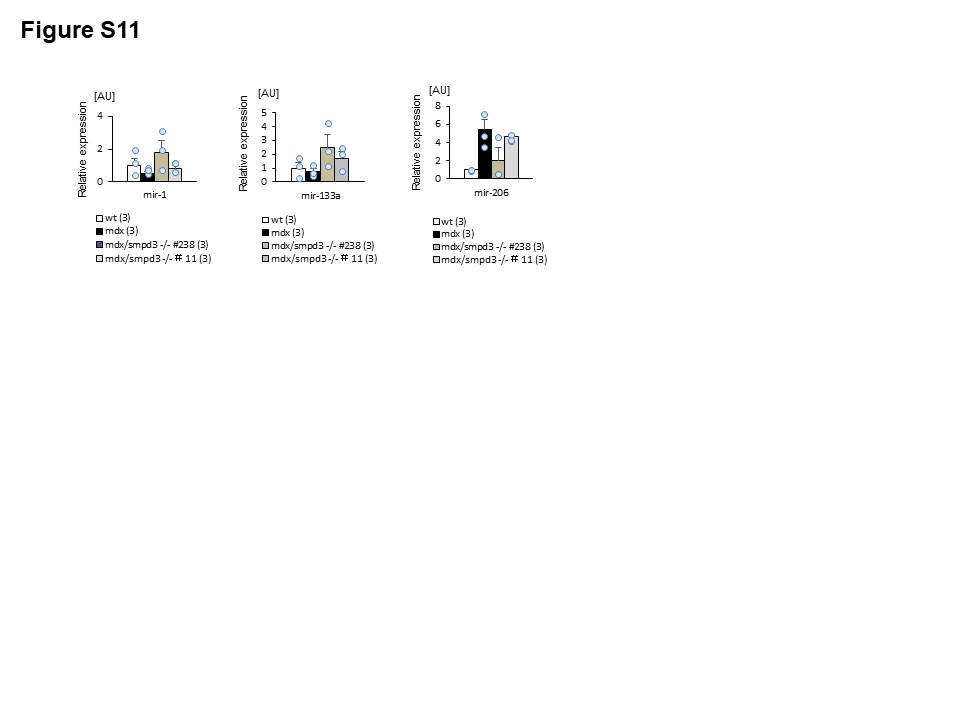

Supplement: Supplementary file 18 — Additional file 18 : Fig. S11. miR-1, miR-133a, and miR-206 levels in the GAS of wt, mdx, mdx:Smpd3−/− (#11 and #238) mice at 12 weeks of age were analyzed using real-time qPCR. [file 12916_2020_1805_MOESM18_ESM.jpg]

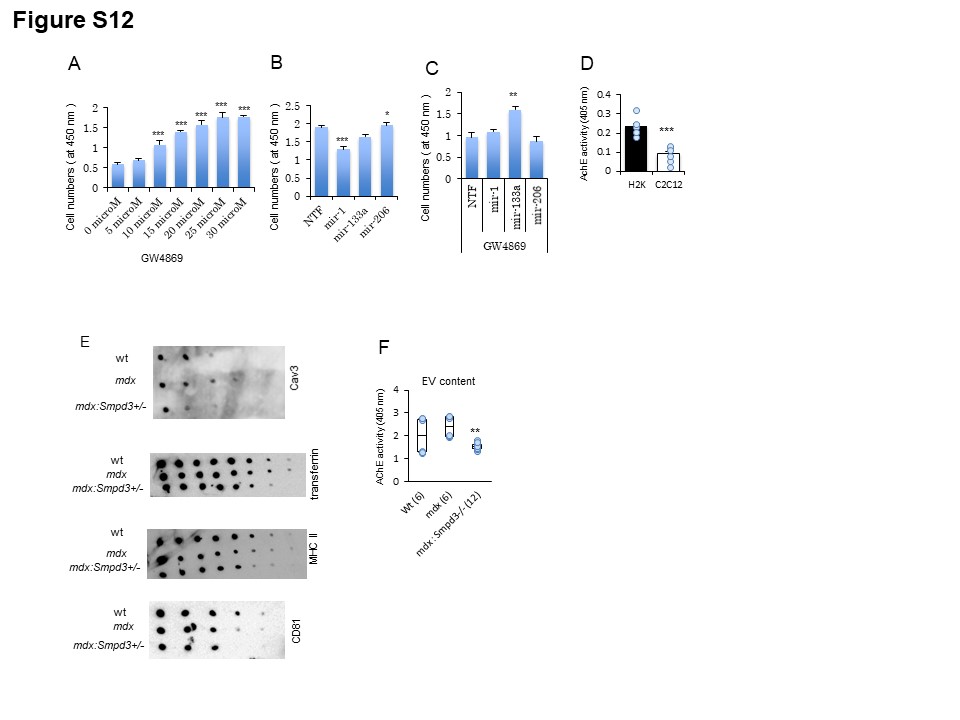

Supplement: Supplementary file 19 — Additional file 19 : Fig. S12. (A) H2K myotubes differentiated for three days were incubated with or without GW4869 (5, 10, 15, 20, 25, and 30 μM) in serum-depleted medium for 24 h. (B, C) H2K myotubes differentiated for six days and transfected with miR-1, miR-133a, and miR-206 were incubated in serum-depleted medium with or without GW4869 (25 μM) for four days. (D) Extracellular vesicles (EVs) were extracted and quantified based on acetylcholinesterase (AChE) activity from H2K and C2C12 cells. Data presented are the mean + standard error (SE) of absorbance at 450 nm of CCK-8. Each independent experiment was repeated at least three times. (E) EV content of the sera of 12-week-old wt, mdx, and mdx:Smpd3+/− mice. (F) EVs were extracted from the sera of wt (n = 6), mdx (n = 6), and mdx:Smpd3−/− mice (n = 12) and quantified based on AChE activity. * p < 0.05, ** p < 0.01. [file 12916_2020_1805_MOESM19_ESM.jpg]

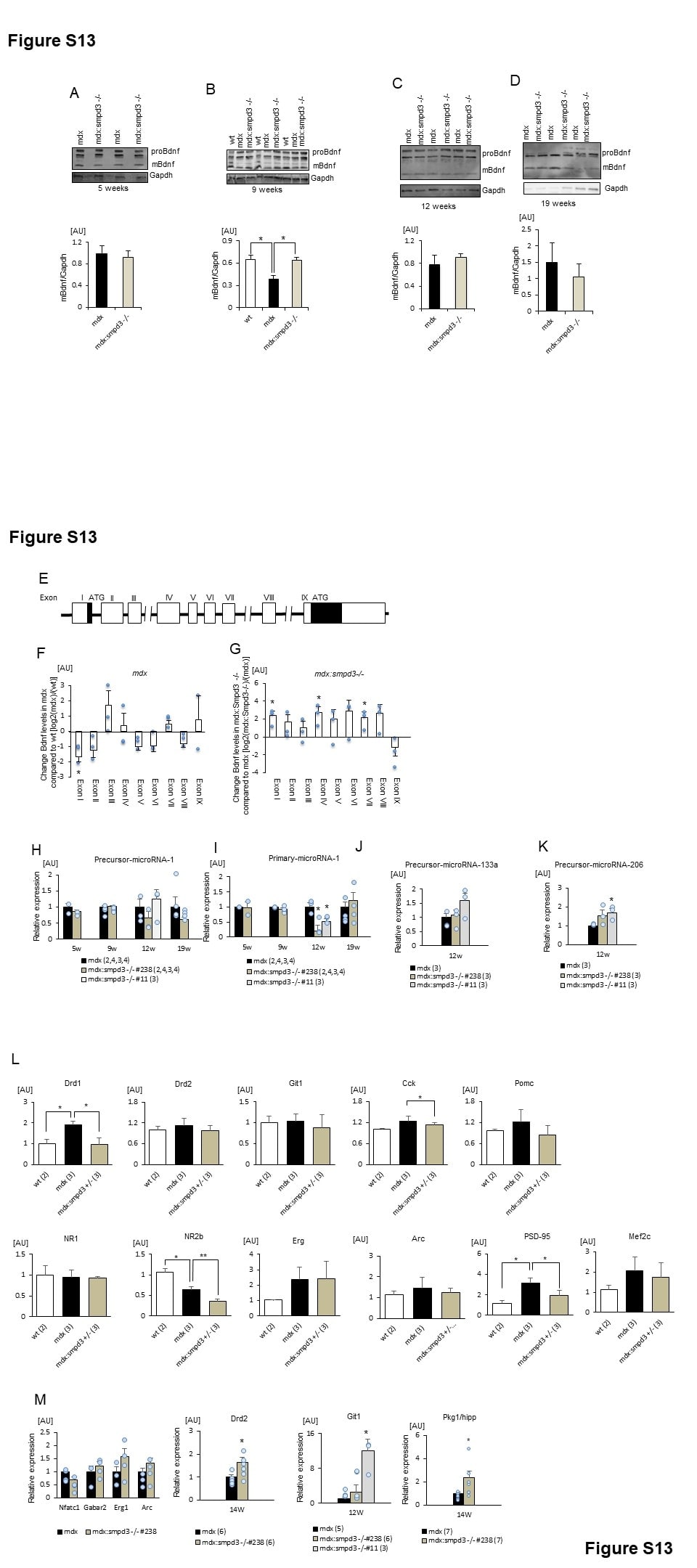

Supplement: Supplementary file 20 — Additional file 20 : Fig. S13. Expression analysis in the hippocampus of mdx:Smpd3−/− mice. (A) Representative western blot analysis (top) of whole hippocampal protein homogenates from wt, mdx, and mdx:Smpd3−/− mice at 5 weeks (A), 9 weeks (B), 12 weeks (C), and 19 weeks (D) of age using anti-Bdnf and anti-Gapdh antibodies. The ratio of mBdnf/Gapdh is expressed as arbitrary units [AU] (bottom). (E–G) Expression of Bdnf isoforms in the hippocampi of wt, mdx, and mdx:Smpd3−/− mice at 12 weeks of age based on real-time RT-PCR. (E) Exon/intron structure and alternative transcripts of the mouse Bdnf gene. Exons are indicated as boxes and introns are indicated as lines. Filled black regions in the boxes indicate the translated regions, in which ATG represents the translated start codon. The expression levels of each Bdnf isoform from exon I to exon IX are shown as log2 (fold-changes) in mdx mice relative to wt mice (F) and in mdx:Smpd3−/− mice relative to mdx mice (G). The levels of four myomiRs, precursor-microRNA-1 (H), primary-microRNA-1 (I), precursor-microRNA-133a (J), and precursor-microRNA-206 (K), were analyzed in the hippocampi of Smpd3−/− (#11 and #238) and mdx mice at 12 weeks of age. (L) Expression of genes induced in the hippocampi of wt, mdx, and mdx:Smpd3+/− mice at 12 weeks of age was measured via real-time RT-PCR (n = 3 per genotype). * p < 0.05, ** p < 0.01. (M) Expression levels of the Nfactc1, Gabar2, Egr1, Arc, Drd2, and Git1 genes in the hippocampi of mdx:Smpd3−/− and mdx mice. * p < 0.05. [file 12916_2020_1805_MOESM20_ESM.jpg]
